# One‐Step Induction of Oogonia‐Like and Pro‐Spermatogonia‐Like Cells From Porcine Pluripotent Stem Cells

**DOI:** 10.1002/advs.78213

**Published:** 2026-10-11

**Authors:** Pengcheng He, Liang Zhong, Tianzhi Chen, Yaxuan Yan, Xinze Chen, Runbo Li, Yingjie Wang, Yadi Teng, Minglei Zhi, Meng Wang, Jinying Zhang, Fan Zhao, Dengfeng Gao, Suying Cao, Jianyong Han

**Affiliations:** ^1^ State Key Laboratory of Animal Biotech Breeding Frontiers Science Center for Molecular Design Breeding (MOE) College of Biological Sciences China Agricultural University Beijing China; ^2^ Hebei Key Laboratory of Basic Medicine for Diabetes Shijiazhuang Second Hospital Hebei China; ^3^ Animal Science and Technology College Beijing University of Agriculture Beijing China; ^4^ State Key Laboratory of Swine and Poultry Breeding Industry College of Animal Science and Technology Sichuan Agricultural University Chengdu Sichuan China

**Keywords:** gametogenesis, oogonia, pluripotent stem cells, porcine, primordial germ cells, pro‐spermatogonia

## Abstract

Pigs share a germ cell regulatory network highly similar to that of humans, yet lack a robust system for inducing and expanding germ cells from pluripotent stem cells, impeding large‐animal gametogenesis research. Here, we established a modular platform capable of efficiently inducing and long‐term expanding porcine germ cell‐like cells directly from pluripotent stem cells. We first developed a one‐step method to induce pro‐spermatogonia‐like cells (activating SOX17) and oogonia‐like cells (activating SOX17/TFAP2C) from porcine pre‐gastrulation epiblast stem cells (pgEpiSCs) harboring a NANOS3‐tdTomato (NT) reporter, via activation of exogenous transcription factors. Single‐cell transcriptome profiling revealed a differentiation trajectory from pgEpiSCs through primordial germ cell‐like cells (PGCLCs) to pro‐spermatogonia/oogonia‐like cells, with concomitant differentiation of gonadal somatic cells. Functional analyses demonstrate that EGF activates core PGC network and is essential for porcine PGCLCs survival in a xenogeneic reconstituted ovary, BMP4 drives NANOS3 and DDX4 expression, and IWR1 enhances germ cell differentiation by suppressing mesodermal programs. Finally, to address the challenge of cell quantity, we established a long‐term expansion module based on FGF2, IWR1, and EGF that sustains NT‐positive cells for up to 120 days while preserving their early PGC and pro‐spermatogonia‐like molecular characteristics. Our study provides new insights into porcine gametogenesis and reproductive development across species.

## Graphical abstract

This study established a one‐step method inducing pro‐spermatogonia‐like (activating SOX17) and oogonia‐like (activating SOX17/TFAP2C) cells from porcine pgEpiSCs with a NANOS3‐tdTomato reporter. EGF activates the core PGC program and supports survival; BMP4 further induces NANOS3 and DDX4 expression; IWR1 enhances PGCLC efficiency by suppressing mesoderm. A long‐term expansion culture system maintains pro‐spermatogonia‐like cell identity for 120 days.

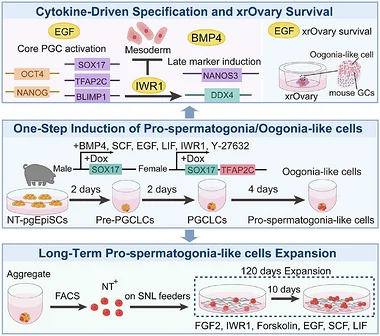

## 1 Introduction

Primordial germ cells (PGCs) are the progenitor cells of sperm and eggs, giving rise to gametes that ensure reproduction, heredity, and evolution of a given species [1]. Pigs closely resemble humans in organ size, embryonic morphology, and possess conserved gamete specification mechanisms, making them an ideal large‐animal model for investigating human gametogenesis [2]. Establishing in vitro gametogenesis in pigs—particularly through the differentiation of pluripotent stem cells (PSCs) into the germ cell lineage—is therefore a key step toward leveraging this model [3]. Beyond basic research, such studies hold promise for applications in human reproductive medicine, elite livestock breeding, and endangered species conservation.

Recent advances have enabled the in vitro reconstitution of germ cell development in mice. Mouse embryonic stem cells (mESCs) can be induced with Activin A and FGF2 to form epiblast‐like cells (EpiLCs), which subsequently give rise to mouse primordial germ cell‐like cells (mPGCLCs) [4]. Upon transplantation into seminiferous tubules or ovaries, these mPGCLCs generate functional gametes; alternatively, aggregation with fetal gonadal somatic cells yields functional oocytes and sperm without the need for an in vivo microenvironment [4, 5, 6, 7]. However, the trajectory of germ cell differentiation differs markedly between humans and mice. Human pluripotent stem cells (hPSCs) require treatment with Activin A and CHIR to generate incipient mesoderm‐like cells (iMeLCs), which then differentiate into human primordial germ cell‐like cells (hPGCLCs) [8, 9, 10]. More critically, the core regulatory networks governing germ cell specification are fundamentally divergent: the mPGC network is driven by *Prdm14*, *Blimp1*, and *Tfap2c*, whereas the hPGC network is dominated by *SOX17*, *TFAP2C*, and *BLIMP1*—a divergence further exemplified by the absence of *SOX2* expression in hPGCLCs [9, 11, 12].

Given the limitations of chemical induction alone, activation of exogenous transcription factors has been shown to substantially enhance PGCLCs differentiation efficiency. In mEpiLCs, activation of PRDM14 alone suffices to induce mPGCLCs, while the addition of TFAP2C and BLIMP1 further improves efficiency [13]. In human iMeLCs, SOX17 activation is essential for hPGCLCs induction, and combining it with TFAP2C and BLIMP1 markedly boosts efficiency [10]. Following hPGCLCs specification, further differentiation into oogonia or pro‐spermatogonia typically requires support from gonadal somatic cells. Although allogeneic reconstituted ovaries and testes have been used for this purpose, their application is constrained by the difficulty of obtaining sufficient source materials. Xenogeneic reconstituted ovaries and testes—generated by aggregating hPGCLCs with mouse gonadal somatic cells—have successfully produced oogonia‐like and pro‐spermatogonia‐like cells, but these systems are characterized by prolonged culture durations and generally low efficiency [14, 15]. While PGCLCs have been successfully induced in cattle and rhinos, further differentiation into oogonia‐like or pro‐spermatogonia‐like cells has not yet been achieved [16, 17]. Importantly, the pig serves a dual role as both a critical translational bridge between rodent models and human reproductive biology, and a valuable large‐animal model for agricultural breeding [2]. Its pre‐implantation embryonic development and gonadal size closely mirror human physiology, making it an indispensable intermediate model for validating findings obtained from mice before translating to human applications; at the same time, as an important livestock species, the pig provides a unique platform for developing in vitro gametogenesis technologies that can accelerate genetic improvement and enhance reproductive efficiency in animal production [10]. Establishing a strategy for the direct differentiation of porcine PSCs into oogonia‐like and pro‐spermatogonia‐like cells and their long‐term expansion could therefore not only offer valuable insights into in vitro gametogenesis in humans and other large animal species, but may also provide a foundation for future functional validation of candidate genes, drug screening, and the development of fertility preservation technologies, with the long‐term goal of serving as a translational bridge from rodent models to human clinical applications.

A central bottleneck in realizing the potential of this platform lies in the scarcity of PGCs and the difficulty of expanding them in vitro. Addressing this requires a dedicated expansion system for PSC‐derived PGCLCs. Robust expansion is not only a prerequisite for dissecting the molecular mechanisms of gametogenesis, but also a critical step toward generating a scalable “seed cell” resource for stem cell‑based breeding—linking in vitro gametogenesis directly to agricultural innovation. In mice, Forskolin and Rolipram have been used to establish an expansion system for mPGCLCs, facilitating the elucidation of key signals and mechanisms governing female sex determination in germ cells [18]. In humans, an expansion culture system centered on Forskolin and FGF2 has enabled approximately 10^6^‐fold expansion of hPGCLCs within four months while preserving their primordial characteristics, including transcriptome, DNA methylation profile, and capacity to differentiate into oogonia‐like or pro‐spermatogonia‐like cells; addition of BMP2 further supports the differentiation and long‑term expansion of human oogonia‑like and pro‑spermatogonia‑like cells [19]. To date, however, no such expansion system has been established for porcine germ cells.

In this study, we established a modular platform for the direct differentiation and long‐term expansion of porcine germ cell‐like cells from pluripotent stem cells. We first developed a one‑step induction system that differentiates porcine pre‐gastrulation epiblast stem cells (pgEpiSCs) directly into pro‑spermatogonia‑like and oogonia‑like cells without intermediate induction steps, using a NANOS3‑tdTomato (NT) reporter combined with activation of exogenous transcription factors [20]. Notably, this system facilitated the co‐differentiation of gonadal somatic cell‐like cells (Sertoli‐like and granulosa‐like cells) alongside germ cells. Through analysis of ligand‐receptor interactions and functional assays, we found that EGF activates the core PGC regulatory network, and BMP4 further drives NANOS3 and DDX4 expression. Furthermore, EGF proved essential for porcine PGCLC survival, thereby overcoming the compatibility limitations associated with xenogeneic reconstituted ovaries (xrOvaries). Finally, to address the bottleneck of cell quantity, we further integrated a long‐term expansion module based on FGF2, IWR1, and EGF that sustains the germ cell‐like cells for at least 120 days while preserving their early PGC or pro‐spermatogonia‐like molecular characteristics. Collectively, this platform not only advances our understanding of porcine gametogenesis but may also provide a foundation for future stem cell‐based breeding strategies and a reference for developing in vitro gametogenesis systems in other large animal species.

## 2 Results

### 2.1 Ectopic Expression of SOX17 Alone in Male pgEpiSCs or Combined With TFAP2C in Female pgEpiSCs Induces pPGCLCs

To investigate the differentiation process and efficiency of porcine primordial germ cells (pPGCs), we constructed a reporter system targeting a pPGC marker gene in porcine pre‐gastrulation epiblast stem cells (pgEpiSCs) (Figure 1A). *NANOS3* was selected due to its evolutionary conservation, critical roles in PGC specification in mice and humans, and specific expression in porcine fetal germ cells (Figure 1A and Figure S1A) [10, 21, 22, 23]. Two independent lines of male and female pgEpiSCs were maintained on feeder cells in 3i/LAF medium. Using CRISPR/Cas9, we isolated homologous recombinants carrying the targeted NANOS3‐2A‐tdTomato (NT) allele (Figure S1B). Selected clones were homozygous for the targeted allele and maintained expression of the core pluripotency factors OCT4, SOX2, and NANOG, as confirmed by immunostaining (Figure S1C–E). These validated lines were used for subsequent experiments.

**FIGURE 1:**
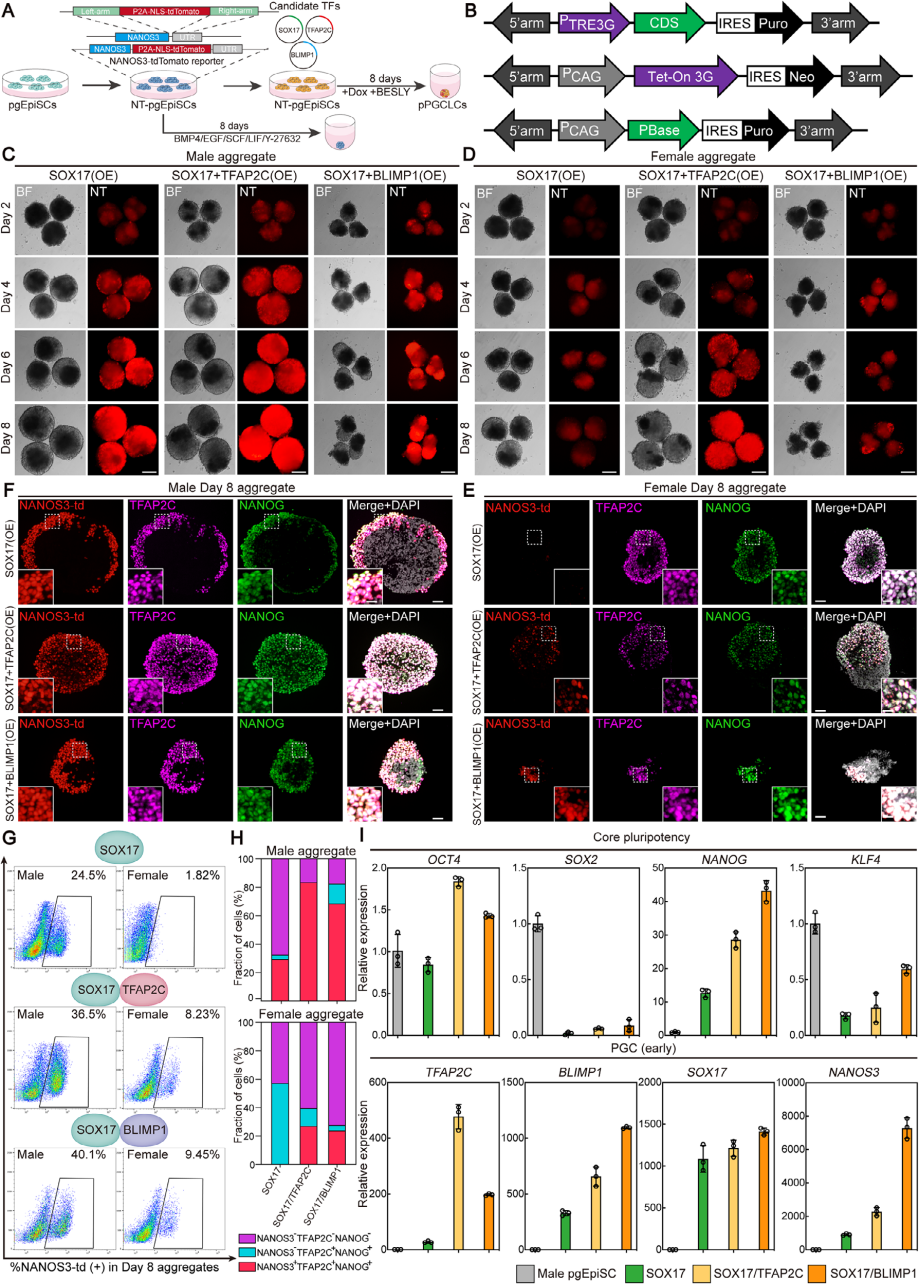
Porcine pgEpiSCs differentiate directly into pPGCLCs through activation of exogenous transcription factors. (A) Schematic diagram of one‐step directed differentiation from pgEpiSCs to pPGCLCs. pgEpiSCs with a NANOS3 reporter system are directly differentiated into pPGCLCs upon activation of exogenous transcription factors. (B) Expression of Dox‐inducible candidate transcription factor (SOX17, TFAP2C and BLIMP1) in NANOS3–tdTomato (NT) reporter pgEpiSCs. (C) Fluorescence microscope images of NT reporter transgenic male pgEpiSCs cultured for 2, 4, 6, and 8 days with induction of SOX17, SOX17/TFAP2C, or SOX17/BLIMP1 in the presence of cytokines (BMP4, EGF, SCF, LIF, Y‐27632; referred to as BESLY). Scale bars: 100 µm. Data from three biologically independent experiments (n = 3) are shown for each condition. (D) Fluorescence microscope images of NT reporter transgenic female pgEpiSCs cultured for 2, 4, 6, and 8 days with induction of SOX17, SOX17/TFAP2C, or SOX17/BLIMP1 in the presence of cytokines (BESLY). Scale bars: 100 µm. Data from three biologically independent experiments (n = 3) are shown for each condition. (E) Immunofluorescence (IF) analyses of TFAP2C (purple) and NANOG (green) expression in NT‑positive cells from aggregates induced from transgenic female pgEpiSCs after 8 days of induction with SOX17, SOX17/TFAP2C, or SOX17/BLIMP1. Cells were counterstained with DAPI (gray); merged images are shown on the right. Scale bars: 50 µm (zoomed‐out), 20 µm (zoomed‐in). (F) IF analyses of TFAP2C (purple) and NANOG (green) expression in NT^+^ (red) cells from aggregates induced from transgenic male pgEpiSCs after 8 days of induction with SOX17, SOX17/TFAP2C, or SOX17/BLIMP1. Cells were counterstained with DAPI (gray); merged images are shown on the right. Scale bars: 50 µm (zoomed‐out), 20 µm (zoomed‐in). (G) Flow cytometry analysis of NT reporter transgenic male and female pgEpiSCs cultured for 8 days with induction of SOX17, SOX17/TFAP2C, or SOX17/BLIMP1 in the presence of cytokines (BESLY). (H) Quantification of the immunostaining data shown in (E) and (F). The numbers of cells with the indicated expression patterns (NANOS3^−^TFAP2C^−^NANOG^−^, NANOS3^−^TFAP2C^+^NANOG^+^, and NANOS3^+^TFAP2C^+^NANOG^+^) induced from transgenic pgEpiSCs after 8 days of induction with SOX17, SOX17/TFAP2C, or SOX17/BLIMP1 are shown. (I) RT–qPCR analysis of gene expression in NT‑positive cells derived from male pgEpiSCs induced for 8 days with SOX17, SOX17/TFAP2C, or SOX17/BLIMP1 transgenes in the presence of cytokines (BESLY). Values are normalized to *GAPDH* and presented as mean ± SD relative to untreated day 0 controls. Data from three biologically independent experiments (n = 3) for day 0 and day 8.

We first examined whether pgEpiSCs could be directly induced into pPGCLCs. NT pgEpiSCs were dissociated into single cells and cultured as aggregates in aRB27 medium (advanced RPMI 1640 supplemented with 1% B27) supplemented with bone morphogenetic protein 4 (BMP4), epidermal growth factor (EGF), stem cell factor (SCF), leukemia inhibitory factor (LIF), and a ROCK inhibitor (Y‐27632) (collectively referred to as BESLY), at a density of 8000 cells per aggregate (Figure 1A) [24, 25]. The aggregates remained NT‐negative from day 0 to day 8; FACS analysis detected only 0.36% and 0.37% NT‐positive cells from male and female pgEpiSCs, respectively, at day 8 (Figure S1F–I). We next analyzed gene expression dynamics during aggregate induction by qPCR. The pluripotency genes *OCT4*, *NANOG*, and *SOX2* declined rapidly, whereas pPGC markers including *SOX17*, *TFAP2C*, *BLIMP1*, and *NANOS3* showed only minimal increases relative to pgEpiSCs (Figure S1J) [22]. These results suggest that pgEpiSCs cannot directly differentiate into pPGCLCs under the tested conditions.

To promote direct differentiation, we introduced exogenous transcription factors to activate the core PGC regulatory network. Given the similarity between porcine and human PGCs, both of which utilize SOX17, TFAP2C, and BLIMP1 as core regulators, we established an inducible expression system for these three genes in the NT reporter line (Figure 1A,B) [26]. Activation of TFAP2C or BLIMP1 alone yielded only 0.97% and 1.05% NT‐positive cells by FACS on day 8, with no significant activation of the PGC core network or downregulation of the pluripotency network as assessed by qPCR, suggesting that individual activation of TFAP2C or BLIMP1 fails to induce pgEpiSC differentiation toward pPGCs (Figure S2A–D). Notably, ectopic expression of SOX17 alone induced NT‐positive cells in male pgEpiSCs but not in female pgEpiSCs (Figure 1C,D). This sex‐specific difference reflects higher expression of PGC core regulators (including *SOX17* and *TFAP2C*) in male pgEpiSCs, which may explain their more robust PGC differentiation (Figure S2E) [20]. Although SOX17 alone failed to induce NANOS3 expression in female cells, immunofluorescence detected both TFAP2C and NANOG, indicating partial activation of the PGC core network (Figure 1E,F). To overcome this limitation in female cells, we combined SOX17 with either TFAP2C or BLIMP1. Both combinations induced NT‐positive female cells, with efficiencies of 8.23% (SOX17/TFAP2C) and 9.45% (SOX17/BLIMP1), though SOX17/BLIMP1 aggregates appeared smaller with poor morphology (Figure 1D,G). In male pgEpiSCs, combining SOX17 with TFAP2C or BLIMP1 significantly increased NT‐positive cells to 36.5% and 40.1%, respectively, with similar morphological abnormalities observed in the SOX17/BLIMP1 condition (Figure 1C,G).

Immunofluorescence confirmed that NT‐positive cells co‐expressed the PGC markers TFAP2C and NANOG, validating reporter specificity (Figure 1E,F and Figure S2F,G). Co‐activation of SOX17 and TFAP2C yielded the highest proportion of NANOS3^+^TFAP2C^+^NANOG^+^ cells (Figure 1H). FACS analysis showed that SOX17 alone induced 24.5% NT‐positive cells in male aggregates vs. 1.82% in female aggregates (Figure 1G). The SOX17/BLIMP1 combination gave slightly higher NT‐positive rates than SOX17/TFAP2C in both sexes, although the morphological quality was poor (Figure 1E,F). These results demonstrate that SOX17 initiates the core PGC network, and combination with TFAP2C or BLIMP1 markedly improves differentiation efficiency, a mechanism conserved in humans. Importantly, NT‐positive cells expressed *OCT4* and *NANOG* but not *SOX2*, with upregulation of *SOX17*, *TFAP2C*, and *NANOS3*, a feature consistent with early porcine PGCs in vivo (Figure 1I and Figure S2H) [27]. In summary, pgEpiSCs cannot be directly induced into pPGCLCs by chemical induction alone; SOX17 alone activates the PGC network and induces pPGCLCs in male cells, whereas female cells require SOX17 combined with TFAP2C or BLIMP1.

### 2.2 WNT Inhibitor IWR1 Enhances pPGCLC Induction Efficiency by Suppressing Mesoderm Differentiation

Given that pgEpiSC maintenance depends on FGF, TGF‐β, and WNT signaling, we tested whether modulating these pathways affects pPGCLCs differentiation efficiency [20, 28]. To further optimize pPGCLC induction, we used male pgEpiSCs with doxycycline (Dox) inducible expression of SOX17. We added FGF2 (an FGF agonist) and PD173074 (an FGF antagonist) individually to the basal induction medium (BMP4, EGF, SCF, LIF, Y‐27632, BESLY). Under these conditions, NT‐positive cells did not exhibit significant changes after 8 days of culture. Interestingly, the addition of Activin A (a TGF‐β agonist) or CHIR (a WNT agonist) also failed to induce NT‐positive cells (Figure 2A and Figure S3A,B). In contrast, various WNT inhibitors effectively enhanced the differentiation efficiency into NT‐positive cells, with IWR1 being the most potent, yielding 45.1% NT‐positive cells (Figure 2A,B). Notably, under the BESLY+IWR1 condition, the expression of pluripotency‐associated genes, including *OCT4* and *NANOG*, was maintained, and rapid upregulation of early PGC marker genes was observed (Figure 2C). This observation is consistent with previous findings that inhibition of WNT signaling enhances PGCLC differentiation in humans [29]. Moreover, under BESLY+IWR1 conditions, not only early PGC genes but also pro‐spermatogonia/oogonia genes, such as *DAZL* and *DDX4*, were effectively activated (Figure 2C) [30]. Immunofluorescence analysis revealed that cells under these conditions expressed pluripotency markers (OCT4, NANOG) along with early PGC markers (SOX17, TFAP2C) and the pro‐spermatogonia/oogonia marker DDX4 (Figure 2D). Taken together, these findings suggest that inhibition of WNT signaling promotes the induction of NT‐positive cells and effectively enhances the upregulation of both early and late PGC‐associated genes.

**FIGURE 2:**
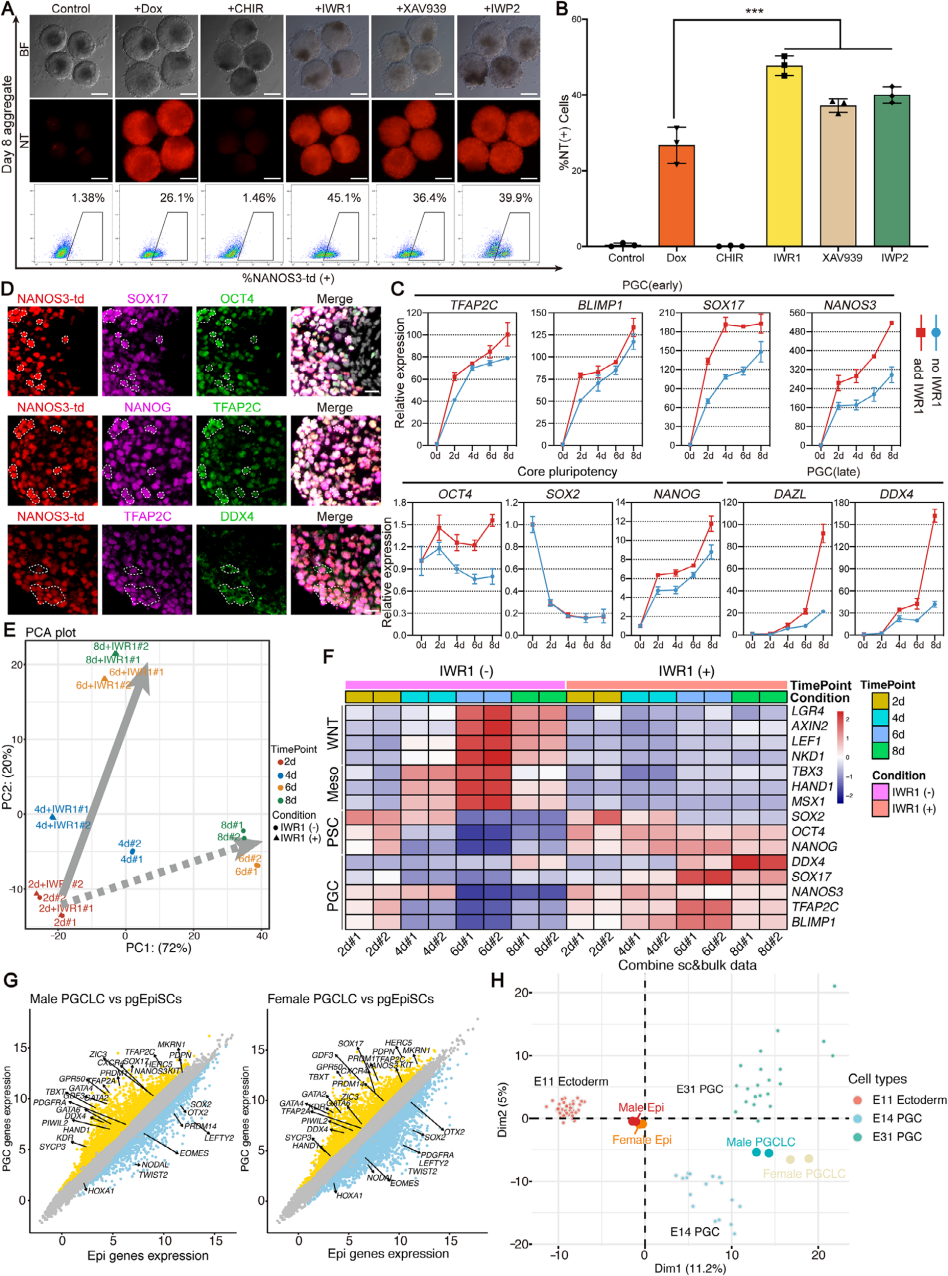
The WNT inhibitor IWR1 effectively enhances the differentiation efficiency of pPGCLCs. (A) Fluorescence microscope images and flow cytometry of NT reporter transgenic male pgEpiSCs cultured for 8 days with induction of SOX17 in the presence of cytokines: control (no cytokines), BESLY, BESLY + CHIR, BESLY + IWR1, BESLY + XAV939, BESLY + IWP2. Scale bars: 100 µm. (B) Bar graph quantification of the percentage of NANOS3‐tdTomato‐positive cells in pgEpiSCs (control, no cytokines) and Day 8 aggregates under the indicated treatments. Statistical significance was determined by *t*‐test: ns, *P* > 0.05, ^*^P < 0.05, ^**^P < 0.01, ^***^P < 0.001. Error bars represent mean ± SEM. Biologically independent experiments, n = 3. (C) RT–qPCR analysis of gene expression in NT‐positive cells derived from male pgEpiSCs induced for 2, 4, 6, and 8 days with SOX17 transgenes in the presence of cytokines (BESLY) with or without IWR1. Values are normalized to *GAPDH* and presented as mean ± SD relative to untreated day 0 controls. Biologically independent experiments, n = 3. (D) IF analyses of the expression of SOX17, OCT4, NANOG, and DDX4 in NT^+^ (red) cells of aggregates induced from transgenic male pgEpiSCs after 8 days induction of SOX17 in the presence of cytokines (BESLY + IWR1). The cells were counterstained with DAPI (gray), and merged images are shown on the right. Scale bars: 50 µm. (E) Principal component analysis (PCA) of global gene expression profiles during PGCLC differentiation. Cells were treated with IWR1 (+, triangles) or untreated (−, circles) at indicated time points (days 2, 4, 6, and 8). Dashed and solid lines indicate the differentiation trajectories under control (BESLY) and IWR1‐treated (BESLY + IWR1) conditions, respectively. (F) Heatmap of lineage‐specific gene expression during pPGCLC differentiation. Genes are grouped by functional lineage (WNT, mesoderm, PSC, PGC). Samples are ordered by time point (2d–8d) and treatment condition (IWR1−/+). Color scale represents relative expression levels. (G) Scatter plots comparing PGC gene expression vs. pluripotency gene expression in male (left) and female (right) PGCLCs relative to pgEpiSCs. Highlighted genes include key PGC markers and pluripotency markers. (H) Combined single‐cell and bulk RNA‐seq PCA, integrating PGCLC differentiation data with in vivo PGC. Clusters represent E11 ectoderm, E14 PGC, E31 PGC, male/female pgEpiSCs, and male/female PGCLCs.

To elucidate the mechanism by which IWR1 promotes pPGCLCs induction, we performed time‐course transcriptome analysis with or without IWR1 (Figure 2E). Samples were collected every two days until day 8 (Figure S3C). Global transcriptome correlation analysis revealed that IWR1 had minimal impact at early stages (days 2–4), but its effect became pronounced at later stages (days 6–8) (Figure 2E and Figure S3C). IWR1 induced a distinct differentiation trajectory compared to the untreated control, with significant divergence observed by days 6 and 8 (Figure 2E and Figure S3C). IWR1 treatment strongly suppressed the expression of canonical WNT target genes, including *LGR4*, *AXIN2*, and *LEF1*, while effectively activating the core PGC regulatory network (Figure 2F) [31]. Additionally, mesoderm‐related genes were significantly downregulated, suggesting that IWR1 may facilitate PGC network activation by suppressing mesoderm‐specific programs (Figure 2F) [32]. We performed differentially expressed gene (DEG) analysis between IWR1‐treated and untreated groups on day 8, followed by GO enrichment analysis (Figure S3D,E and Table S1). The upregulated genes were mainly enriched in functions such as “tissue development”, “epithelium development”, and “cell–cell signaling”, whereas the downregulated genes were significantly enriched in terms related to “blood vessel morphogenesis”, “muscle cell proliferation”, and “WNT signaling pathway” (Figure S3E) [33, 34]. Given that vascular and muscle tissues are primarily derived from the mesoderm, these findings suggest that IWR1 likely enhances pPGCLCs induction efficiency by suppressing the expression of mesoderm‐related genes, including those involved in vascular formation and muscle development.

To characterize day 8 pPGCLCs, which were differentiated in aRB27 basal medium supplemented with BMP4, EGF, SCF, LIF, IWR1, and Y‐27632 (collectively referred to as BESLIY), we compared their transcriptomes with those of pgEpiSCs (Figure 2G and Figure S3F,G). At day 8 of differentiation, early PGC markers (*SOX17, TFAP2C, NANOS3*) were significantly upregulated, accompanied by the induction of late markers, including *DDX4* and *SYCP3*, indicating that this system supports differentiation into PGCs that acquire pro‐spermatogonia/oogonia features (Figure 2G). Consistent with this observation, we compared our data with published single‐cell transcriptomic data from porcine in vivo samples (Figure 2H and Figure S3H,I) [22]. The transcriptional profiles of both male and female pPGCLCs were intermediate between those of early PGCs at E14 and late pPGCs at E31, with a closer resemblance to the late pPGCs (Figure 2H). Collectively, IWR1 contributes to this process by enhancing differentiation efficiency, primarily through the suppression of mesoderm differentiation. The established induction system enables differentiation into pPGCLCs that closely resemble E31 late pPGCs in vivo.

### 2.3 Molecular and Epigenetic Characterization of pgEpiSCs Differentiating Into Pro‐Spermatogonia and Oogonia

In mice and humans, direct induction of pro‐spermatogonia or oogonia from PGCLCs is not achievable with standard differentiation media; instead, it requires aggregation with fetal gonadal somatic cells or specialized culture conditions [5, 14, 15, 24, 35, 36, 37]. In contrast, our system enables direct induction of pro‐spermatogonia‐like and oogonia‐like cells from pgEpiSCs. To investigate the underlying mechanism, we performed single‐cell transcriptome sequencing on female and male pgEpiSCs and differentiating aggregates at days 2, 4, and 8 (Figure 3A–C and Figure S4A,B). Notably, gonadal somatic cells emerged alongside germline differentiation (Figure 3A,B and Figure S4C,D). Unsupervised clustering identified distinct somatic populations: Sertoli cells‐like (*SOX9* ^+^) were enriched in cluster 7 of male samples, while granulosa cells‐like (*WNT6* ^+^) were enriched in cluster 5 of female samples (Figure 3D–G and Table S2) [23]. This concomitant differentiation of gonadal somatic cells may be critical for enabling direct induction of pro‐spermatogonia or oogonia in our system.

**FIGURE 3:**
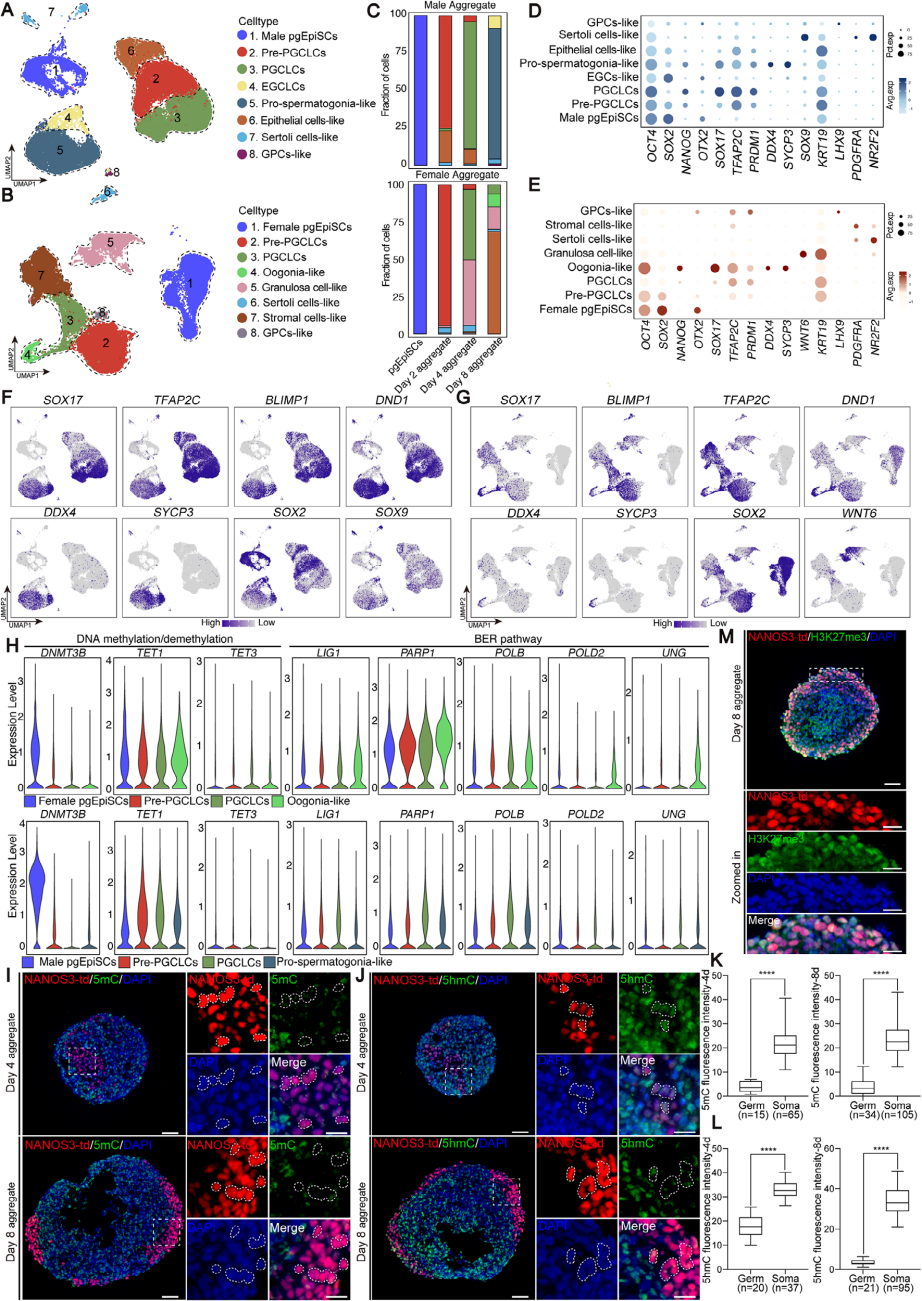
Molecular and Epigenetic Characterization of Pro‐Spermatogonia‐like and Oogonia‐like Cells. (A) UMAP visualization of cell populations from male pgEpiSCs and those induced for 2, 4, and 8 days with SOX17 transgenes in the presence of cytokines (BMP4/EGF/SCF//LIF/IWR1/Y‐27632, BESLIY). Cells are colored by annotated cell types. Embryonic germ cell‐like cells (EGCLCs); Gonadal progenitor cell‐like (GPC‐like). (B) UMAP visualization of cell populations from female pgEpiSCs and those induced for 2, 4, and 8 days with SOX17/TFAP2C transgenes in the presence of cytokines (BESLIY). Cells are colored by annotated cell types. Gonadal progenitor cell‐like (GPC‐like). (C) Bar charts showing the fraction of each cell type in male (top) and female (bottom) pPGCLCs at different developmental stages (pgEpiSCs, Day 2, Day 4, and Day 8). (D, E) Dot plots displaying the expression of key marker genes across male (D) and female (E) cell type clusters, with dot size representing the percentage of cells expressing the gene and color intensity representing expression level. (F) UMAP visualization of representative genes for male PGC and gonadal somatic cells. Expression levels were color–coded. (G) UMAP visualization of representative genes for female PGC and gonadal somatic cells. Expression levels were color–coded. (H) Violin plots showing the expression levels of genes involved in DNA methylation/demethylation and base excision repair (BER) pathway across four female cell types: pgEpiSCs, Pre‐PGCLCs, PGCLCs, and Oogonia‐like (Top). Violin plots displaying the expression of the same set of epigenetic and DNA repair genes across male cell populations: pgEpiSCs, Pre‐PGCLCs, PGCLCs, and Pro‐spermatogonia‐like (Bottom). (I, J) IF analysis of (I) 5mC (green) and (J) 5hmC (green) expression in NT^+^ cells (red) from aggregates derived from male pgEpiSCs induced with SOX17 transgenes for 4 and 8 days in medium containing BESLIY. Nuclei were counterstained with DAPI (blue). Scale bars: 50 µm (zoomed‐out), 20 µm (zoomed‐in). (K, L) Box plots quantifying the fluorescence intensity of (K) 5mC (top) and (L) 5hmC (bottom) in germ cells (Germ) and somatic cells (Soma) at Day 4 (4d) and Day 8 (8d). Sample sizes (n) are indicated for each group. *t*‐test: ^*^ ^***^P < 0.0001. Error bars represent mean ± SEM. (M) IF analysis of H3K27me3 (green) expression in NT^+^ cells (red) from aggregates derived from male pgEpiSCs induced with SOX17 transgenes for 4 and 8 days in medium containing BESLIY. Nuclei were counterstained with DAPI (blue). Scale bars: 50 µm (zoomed‐out), 20 µm (zoomed‐in).

We next investigated the differentiation trajectory from pgEpiSCs to pro‐spermatogonia‐like and oogonia‐like cells. Unsupervised clustering revealed a continuum of cell states. pgEpiSCs (Cluster 1) expressed core pluripotency factors (*OCT4*, *SOX2*, *OTX2*). Pre‐PGCLCs (Cluster 2) retained *SOX2* and gained *TFAP2C* (Figure 3D,E and Figure S4E,F and Table S2). PGCLCs (Cluster 3) maintained *OCT4* and *NANOG* but downregulated *SOX2* and acquired *SOX17*, *TFAP2C*, and *BLIMP1* (Figure 3F,G and Table S2). Pro‐spermatogonia‐like cells (Cluster 5, male) and oogonia‐like cells (Cluster 4, female) activated late germ cell markers including *DDX4* and *SYCP3* (Figure 3F,G and Table S2). Significantly more pro‐spermatogonia‐like cells were obtained from male pgEpiSCs than oogonia‐like cells from female pgEpiSCs. These transcriptional signatures define a continuous differentiation trajectory from pgEpiSCs through Pre‐PGCLCs and PGCLCs to pro‐spermatogonia‐like and oogonia‐like cells.

To investigate key biological events during germline differentiation, we performed DEG analysis across sequential stages (Figure S5A,B). GO enrichment analysis revealed stage‑specific biological processes. Pro‑spermatogonia‑like and oogonia‑like cells were enriched for terms related to “germ cell development”, and female cells additionally showed enrichment for the “meiotic cell cycle”, reflecting progressive acquisition of germ cell fate (Figure S5A,B and Table S3). Furthermore, gonadal somatic cells emerged alongside germ cell differentiation (day 2–8), with immunofluorescence showing NT‐positive germ cells in the aggregate outer region and SOX9^+^/KRT19^+^ somatic cells in the inner region (Figure S5C,D). GO enrichment revealed Sertoli cell‐like enriched in “male gonad development” and “sex differentiation”, and granulosa cell‐like enriched in “response to hormone” and “steroid metabolic process” (Figure S5E,F and Table S3). Together, these findings suggest that this system recapitulates key elements of the embryonic gonad niche by concurrently generating germ cells and gonadal somatic‐like cells.

Epigenetic reprogramming in germ cells involves DNA demethylation and histone modifications. Active DNA demethylation is driven by TET enzymes (*TET1, TET2, TET3*), which oxidize 5‐methylcytosine (5mC) to 5‐hydroxymethylcytosine (5hmC), coupled with TDG‐mediated base excision repair (BER) [38, 39]. We analyzed expression dynamics of DNA methylation and histone modification regulators during pro‐spermatogonia‐like and oogonia‐like cell differentiation (Figure 3H and Figure S5G,H). During oogonia‐like differentiation, *DNMT3B* decreased while *TET1* and *TET2* increased, and the BER pathway, previously reported to be upregulated in late porcine PGCs in vivo, was similarly upregulated (Figure 3H). Notably, during male differentiation, BER enrichment was confined to the PGCLC stage and declined in pro‐spermatogonia‐like cells (Figure 3H and Figure S5G,H). This contrasts with the persistently high BER activity in oogonia‐like cells, suggesting that pro‐spermatogonia‐like cells may partially re‐establish DNA methylation, consistent with the conserved pattern in mice and humans (Figure 3H) [40]. To assess DNA demethylation dynamics, we performed immunofluorescence staining, which revealed that NT‐positive germ cells lost 5mC by day 4 with sustained 5hmC expression, whereas by day 8, 5hmC was also largely undetectable (Figure 3I,J). Throughout differentiation, NT‐positive cells maintained significantly lower DNA methylation than surrounding somatic cells (Figure 3K,L). Additionally, immunofluorescence revealed that the repressive histone mark H3K27me3 colocalized with NT‐positive cells, while PRC2 core components (*EED*, *SUZ12*, *RBBP7*) were highly expressed in pPGCLCs. During differentiation into pro‐spermatogonia‐like and oogonia‐like cells, subunits of the antagonizing SWI/SNF complex (*SMARCA5*, *HLTF*) were progressively upregulated (Figure 3M and Figure S5G,H) [41]. Collectively, these findings recapitulate key epigenetic reprogramming events, including DNA demethylation and histone modifications, that occur during porcine germ cell development in vivo.

### 2.4 Comparison of In Vitro‐Derived Germ Cells With Porcine and Human Fetal Germ Cells

To investigate the similarity between in vitro‐derived germ cells and their in vivo counterparts, we first compared female pgEpiSC‐derived pPGCLCs and oogonia‐like cells with porcine E30–E35 female fetal germ cells (RA‐responsive oogonia) [23]. Integrative analysis showed that pPGCLCs and oogonia‐like cells clustered closely with in vivo RA‐responsive oogonia, and pseudotime analysis placed the oogonia‐like cells at an intermediate stage between early pPGCLCs and RA‐responsive oogonia. Both populations ″expressed early PGC markers (TFAP2C, *NANOS3*, *OCT4*) and piRNA pathway‐related genes (*GTSF1*, *HENMT1*, *PLD6*), and also initiated expression of *DDX4* and *SYCP3*, albeit at lower levels than those in RA‐responsive oogonia (Figure 4A–C and Figure S6A,B) [42, 43, 44]. However, *STRA8* and *ZGLP1* were not activated in oogonia‐like cells, accounting for their higher early PGC marker expression (*OCT4*, *NANOG*, *SOX17*, *TFAP2C*) and lower late PGC marker expression (*DDX4*, *SYCP3*, *DPPA5*) relative to RA‐responsive oogonia (Figure 4C) [45]. These results suggest that oogonia‐like cells correspond to an intermediate stage between early pPGCLCs and RA‐responsive oogonia. We next applied the same strategy to male cells, comparing male pgEpiSC‐derived pPGCLCs and pro‐spermatogonia‐like cells with male porcine E30–E35 fetal pro‐spermatogonia (Figure 4D–F and Figure S6C,D). In vivo pro‐spermatogonia clustered closely with pro‐spermatogonia‐like cells, and pseudotime analysis placed the pro‐spermatogonia‐like cells at an intermediate stage between early pPGCLCs and in vivo pro‐spermatogonia. Compared to pPGCLCs, pro‐spermatogonia‐like cells showed gradual downregulation of early PGC markers (*NANOG*, *OCT4*, *TFAP2C*, *NANOS3*) and, like in vivo pro‐spermatogonia, expressed late PGC markers (*DDX4*, *SYCP3*, *PIWIL2*) and spermatogenesis‐related genes (*TERB1*, *SMC3*, *RAD21*), suggesting that pro‐spermatogonia‐like cells partially recapitulate the in vivo pro‐spermatogonia program. (Figure 4F) [46, 47, 48]. Gene‐set scoring and pseudobulk log_2_ fold‐change concordance analyses further showed that the female in vitro transition exhibited statistically significant concordance with the in vivo transition, whereas the male transition exhibited only partial concordance (Figure S6E–N and Table S4).

**FIGURE 4:**
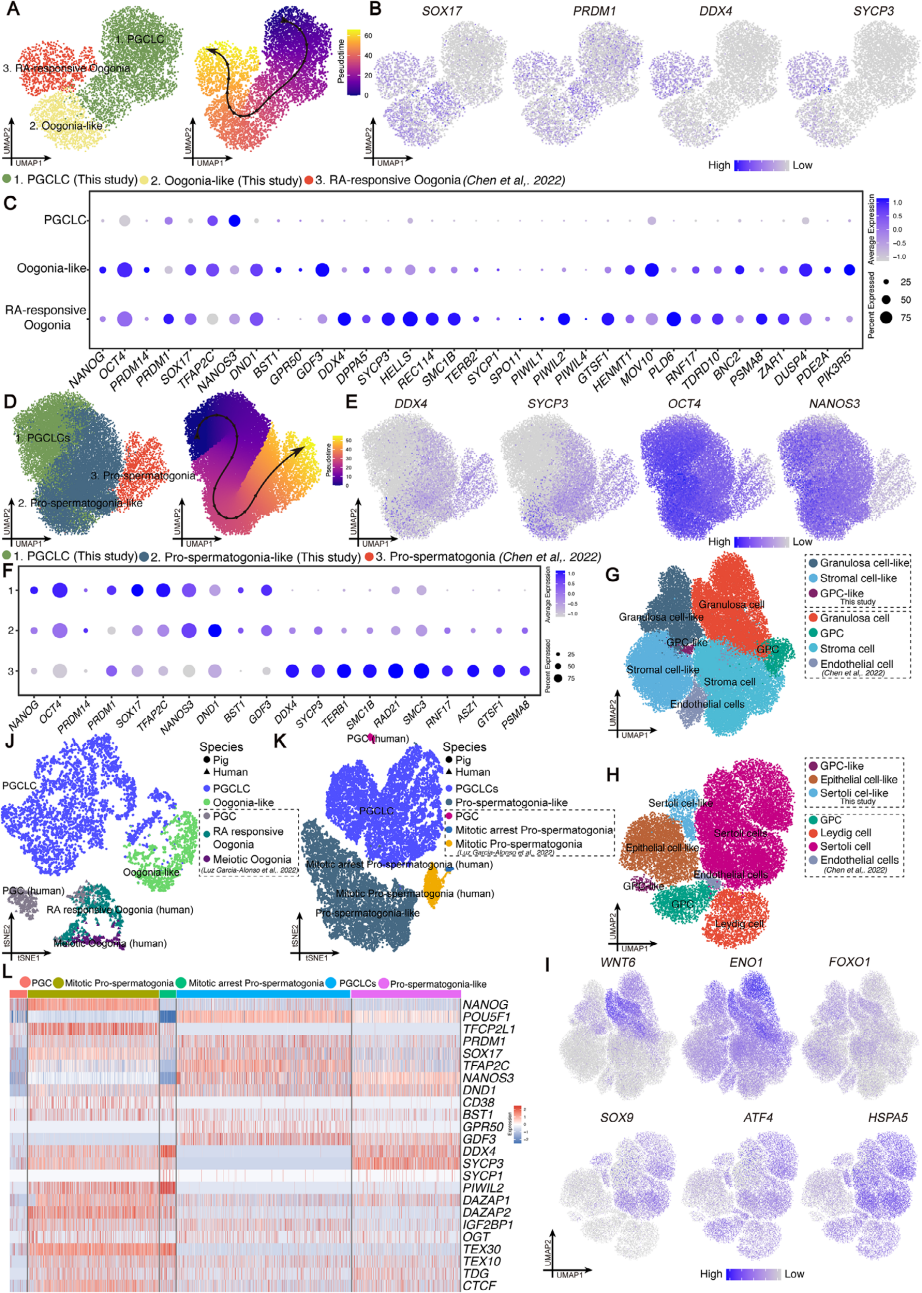
Molecular and Epigenetic Characterization of Pro‐Spermatogonia‐like and Oogonia‐like Cells. (A) Integrative analysis of female pgEpiSC‐derived PGCLCs and oogonia‐like cells with porcine fetal RA‐responsive oogonia, showing UMAP of integrated populations (colored by cell type) and pseudotemporal trajectory from PGCLCs through oogonia‐like cells to RA‐responsive oogonia. (B) UMAP of representative genes for female PGCLCs, oogonia‐like cells, and porcine fetal RA‐responsive oogonia, with expression levels color‐coded. (C) Dot plot of key marker genes across the same female cell types, with dot size indicating expression percentage and color intensity indicating expression level. (D) Integrative analysis of male pgEpiSC‐derived PGCLCs and pro‐spermatogonia‐like cells with porcine fetal pro‐spermatogonia, showing UMAP of integrated populations and pseudotemporal trajectory from PGCLCs through pro‐spermatogonia‐like cells to in vivo pro‐spermatogonia. (E) UMAP of representative genes for male PGCLCs, pro‐spermatogonia‐like cells, and porcine fetal pro‐spermatogonia. (F) Dot plot of key marker genes across the same male cell types. (G) UMAP of integrated female pgEpiSC‐derived granulosa‐like, stromal‐like, and gonadal progenitor‐like cells with porcine fetal granulosa, stromal, gonadal progenitor, and endothelial cells, colored by cell type. (H) UMAP of integrated male pgEpiSC‐derived Sertoli‐like, epithelial‐like, and gonadal progenitor‐like cells with porcine fetal Sertoli, Leydig, gonadal progenitor, and endothelial cells. (I) UMAP of representative genes for granulosa, stromal, and Sertoli cells. (J) tSNE of integrated male PGCLCs and pro‐spermatogonia‐like cells with human fetal germ cells (PGCs, Mitotic Pro‐spermatogonia, Mitotic arrest Pro‐spermatogonia), colored by cell type. (K) tSNE of integrated female PGCLCs and oogonia‐like cells with human fetal germ cells (PGCs, RA‐responsive oogonia, meiotic oogonia). (L) Heatmap of germ lineage‐specific genes in male PGCLCs and Pro‐spermatogonia‐like cells vs. human fetal germ cell subtypes. Sample ordering: porcine PGCLCs, pro‐spermatogonia‐like cells, human PGCs, Mitotic Pro‐spermatogonia, and Mitotic arrest Pro‐spermatogonia. Color scale: relative expression levels.

To further validate the identity of the somatic cell populations that emerged alongside germ cell differentiation, we performed integrative analyses comparing the somatic cells from both female and male differentiation systems with porcine fetal gonadal somatic reference datasets (Figure 4G,H and Figure S7A,B) [23]. The female‐derived granulosa‐like cells, stromal‐like cells, and gonadal progenitor‐like cells, as well as the male‐derived Sertoli‐like cells, epithelial‐like cells, and gonadal progenitor‐like cells, all clustered closely with their corresponding in vivo counterparts in the UMAP embedding. Specifically, granulosa‐like cells highly expressed lineage marker genes such as *WNT6*, *ENO1*, and *SOD1*, while Sertoli‐like cells and in vivo Sertoli cells co‐expressed *SOX9*, *FOS*, *ATF4*, and other genes closely associated with Sertoli cell development (Figure 4I and Figure S7C,D) [23]. These results suggest that the in vitro‐differentiated gonadal somatic cells exhibit expression features similar to those of their in vivo equivalents.

Given the high similarity between porcine and human germ cells, we performed cross‐species comparisons with human fetal germ cells (Figure 4J,K). For male cells, human fetal germ cells (12, 13, and 15 PCW) were classified into PGCs, Mitotic Pro‐spermatogonia, and Mitotic‐arrest Pro‐spermatogonia (Figure 4K) [49]. Human PGCs aligned with porcine PGCLCs, human Mitotic Pro‐spermatogonia with porcine pro‐spermatogonia‐like cells. Core germ cell markers (*OCT4*, *NANOG*, *SOX17*, *TFAP2C*, *NANOS3*) were shared across early stages, while *DDX4* and *SYCP3* were progressively upregulated in both human Mitotic/Mitotic‐arrest Pro‐spermatogonia and porcine pro‐spermatogonia‐like cells, confirming their advanced differentiation state (Figure 4L). Notably, *TDG* and *CTCF* were highly expressed in male germ cells of both species, suggesting conserved epigenetic regulation (Figure 4L) [50, 51]. For female cells, human fetal germ cells (10, 11, 12, 13 PCW) were classified into PGCs, RA‐responsive oogonia, and meiotic oogonia (Figure 4J). Human PGCs clustered with porcine PGCLCs, both expressing early PGC markers (*BLIMP1*, *TFAP2C*, *NANOS3*), while RA‐responsive oogonia activated *STRA8* and *ZGLP1*, and meiotic oogonia subsequently expressed meiotic markers such as *SPO11* and *SYCP1* (Figure S7E). Porcine oogonia‐like cells were positioned between human PGCs and RA‐responsive oogonia, exhibiting elevated late PGC markers but lacking *STRA8* and *ZGLP1*, consistent with an intermediate stage between PGCs and RA‐responsive oogonia. Across both sexes, human germ cells expressed *TFCP2L1*, whereas porcine cells expressed the species‐specific markers *GDF3* and *GPR50* (Figure 4L and Figure S7E) [22]. These cross‐species comparisons suggest that the in vitro‐derived germ cells recapitulate key aspects of the stepwise molecular progression of in vivo germ cell development while maintaining species‐specific marker expression.

In summary, the in vitro‐derived germ and gonadal somatic cells are transcriptionally similar to their in vivo porcine counterparts, and cross‐species comparisons confirm that the germ cells share conserved features with human fetal germ cells while retaining pig‐specific marker expression.

### 2.5 EGF and BMP4 Cooperatively Drive pPGCLCs Differentiation

Gonadal somatic cells play a critical role in the differentiation of PGCs into pro‐spermatogonia and oogonia [52]. Given the concomitant differentiation of gonadal somatic cells during pro‐spermatogonia/oogonia induction, we investigated their potential role in germ cell differentiation by analyzing ligand‐receptor interactions between germ cells and gonadal somatic cells at different time points (Figure 5A–C and Figure S8A,B). Ligand‐receptor interaction analysis revealed distinct signaling dynamics during the differentiation of pro‐spermatogonia‐like and oogonia‐like cells. Male differentiation was characterized by neural (NEGR1‐NEGR1) and growth factor (HBEGF‐EGFR) mediated crosstalk between Sertoli cell‐like (SCL) and pro‐spermatogonia‐like cells (Pro‐SLCs) (Figure 5A, Figure S8A and Table S5). In contrast, female differentiation was dominated by BMP signals from granulosa cell‐like (GCL) and stromal‐like cells (StCLC), with additional contributions from WNT and stroma‐related pathways (Figure 5B and Figure S8B). To further characterize the dynamics of these signaling pathways, we performed signaling activity analysis (Figure 5D,E and Figure S8C,D). This analysis revealed that CDH, NCAM, BMP, and EGF pathways exhibited higher activity in oogonia‐like cells (OLC), whereas in males, BMP and EGF signaling pathways were predominantly enriched in pPGCLCs (Figure 5D,E). Since BMP4 and EGF were present in the differentiation medium, these findings suggest that BMP and EGF signaling may be critical for germ cell differentiation and survival.

**FIGURE 5:**
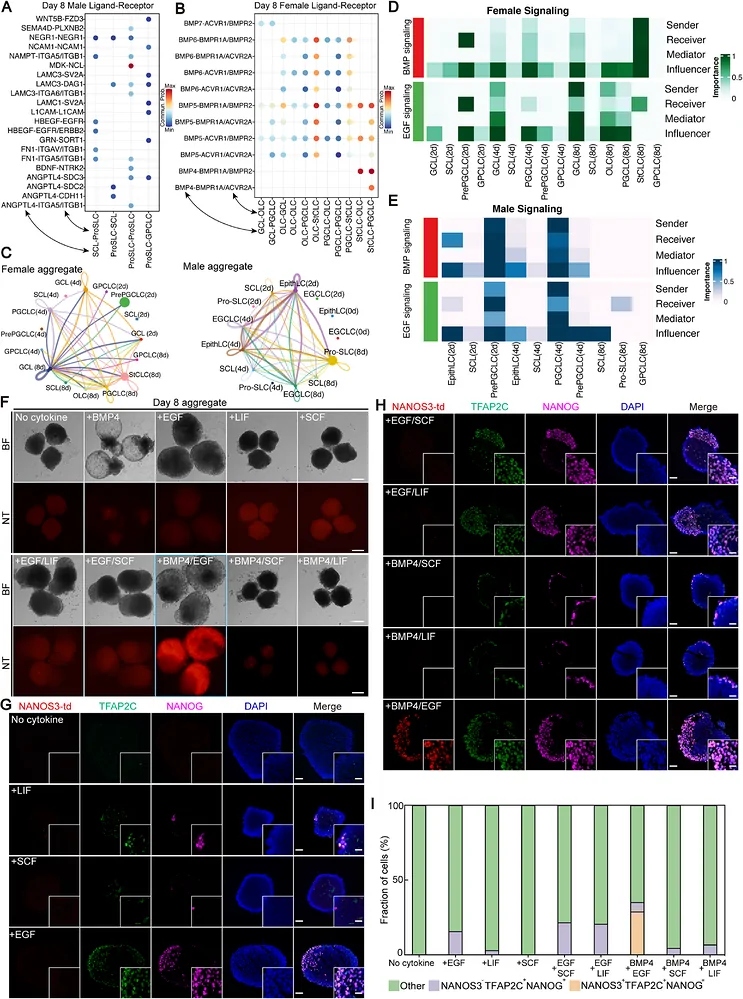
Functional analysis of BMP4 and EGF in germ cell differentiation. (A, B) Dot plots showing ligand–receptor interactions in Day 8 (A) male and (B) female aggregates. Each dot represents a ligand–receptor pair, with color intensity indicating expression level. (C) Cell–cell communication networks in female (left) and male (right) aggregates. Nodes represent cell clusters, and edges represent ligand–receptor interactions. Colors distinguish different cell lineages. (D, E) Heatmaps summarizing the importance of BMP and EGF signaling components in (D) female and (E) male aggregates. Color intensity reflects the relative importance score of each cell type in mediating pathway activity. (F) Fluorescence microscope images of NT reporter transgenic male pgEpiSCs cultured for 8 days with induction of SOX17 in the presence of cytokines: no cytokines, +BMP4, +EGF, +LIF, +SCF, +EGF/LIF, +EGF/SCF, +BMP4/EGF, +BMP4/SCF, and + BMP4/LIF, Scale bars: 100 µm. (G) IF analyses of the expression of TFAP2C (green) and NANOG (purple) in NT^+^ (red) cells of aggregates induced from transgenic male pgEpiSCs after 8 days induction of SOX17 in the presence of cytokines: No cytokine, LIF, SCF, and EGF. The cells were counterstained with DAPI (blue), and merged images are shown on the right. Scale bars: 50 µm (zoomed‐out), 20 µm (zoomed‐in). (H) IF analyses of the expression of TFAP2C (green) and NANOG (purple) in NT^+^ (red) cells of aggregates induced from transgenic male pgEpiSCs after 8 days induction of SOX17 in the presence of cytokines: EGF/SCF, EGF/LIF, BMP4/SCF, BMP4/LIF, and BMP4/EGF. The cells were counterstained with DAPI (blue), and merged images are show on the right. Scale bars: 50 µm (zoomed‐out), 20 µm (zoomed‐in). (I) Quantification of immunostaining data in Figure 5G,H. The numbers of Other, NANOS3^−^TFAP2C^+^NANOG^+^ and NANOS3^+^TFAP2C^+^NANOG^+^ induced from transgenic pgEpiSCs with 8 days induction of SOX17 in the presence of cytokines: no cytokines, +EGF, +LIF, +SCF, +EGF/LIF, +EGF/SCF, +BMP4/EGF, +BMP4/SCF, and +BMP4/LIF.

To elucidate the roles of cytokines in germ cell differentiation, we screened these factors individually or in combination and assessed their effects using the NT reporter system (Figure 5F). Treatment with individual cytokines, including BMP4, EGF, SCF and LIF, failed to induce NT‐positive cells (Figure 5F). Notably, BMP4 alone led to the formation of abnormal cystic structures within aggregates and did not activate the core PGC regulatory network (Figure 5F). Combination screening revealed that only the BMP4 and EGF combination induced NT reporter expression. EGF/LIF and EGF/SCF treatments generated asymmetric structures, whereas BMP4/SCF and BMP4/LIF treatments yielded smaller aggregates without NT‐positive cells, indicating that both EGF and BMP4 are essential for pPGCLCs induction (Figure 5F). To determine their respective effects on germ cell fate acquisition in the context of exogenous SOX17 activation, we performed immunofluorescence staining for the early PGC markers TFAP2C and NANOG. Activating SOX17 alone (without exogenous cytokines) failed to induce TFAP2C and NANOG double‐positive cells (Figure 5G). However, EGF treatment upon SOX17 activation generated TFAP2C and NANOG double‐positive cells, suggesting that EGF can effectively initiate part of the core PGC regulatory network (Figure 5G). In contrast to SCF, which plays a key role in hPGCLCs survival, EGF may serve a more important function in supporting pPGCLCs survival [19, 53, 54]. Further addition of LIF or SCF to EGF treatment increased the proportion of double‐positive cells, yet NT‐positive cells were still not generated (Figure 5H,I). *NANOS3*, a key regulator of epigenetic reprogramming, is essential for PGCs differentiation into oogonia or pro‐spermatogonia [55]. Notably, only the combination of BMP4 and EGF induced NANOS3/TFAP2C/NANOG triple‐positive cells, whereas BMP4/SCF or BMP4/LIF combinations failed to activate the complete core PGC regulatory network (Figure 5H). These findings indicate that, following EGF treatment, BMP4 is essential for activating the complete core PGC regulatory network, including NANOS3 expression.

### 2.6 Dose‐Dependent Roles of EGF and BMP4 in Germ Cell and Somatic Cell Differentiation

Given that EGF and BMP4 are indispensable for pPGCLCs differentiation, we next investigated the effects of different concentrations of these cytokines on germ cell and gonadal somatic cell specification. We added EGF (0, 20, 50, 100, or 200 ng/mL) to the differentiation medium (containing BMP4, SCF, LIF, IWR1, and Y‐27632) and assessed NT‐positive cells (Figure 6A). NT‐positive cells were barely detectable in the control condition (without EGF). Low‐dose EGF (20–50 ng/mL) significantly induced NT‐positive cells, whereas high‐dose EGF (100–200 ng/mL) enlarged the aggregates but appeared to reduce the proportion of NT‐positive cells relative to lower concentrations (Figure 6A). Immunofluorescence staining further confirmed that the proportion of NANOS3/TFAP2C/NANOG triple‐positive cells was highest in the 20 ng/mL EGF group, whereas the abundance of other cell types significantly increased in the high‐dose groups (Figure 6B,C). To investigate whether high EGF concentrations promote gonadal somatic cell differentiation, we performed immunofluorescence staining for somatic markers. Sertoli‐like cells were identified by co‐staining for SOX9 and GATA4, whereas gonadal somatic progenitor‐like cells were marked by KRT19 and GATA4 (Figure S9A) [49, 56]. In the 50 ng/mL EGF treatment group, the proportion of SOX9^+^GATA4^+^ double‐positive cells was highest, reaching approximately 10%; as EGF concentration increased, this proportion gradually declined, while the proportion of KRT19^+^GATA4^−^ cells markedly increased (Figure S9B–D). These findings suggest that high concentrations of EGF may promote the differentiation of KRT19^+^ epithelial‐like cells, which lack GATA4 expression [57]. We further tested the effects of different concentrations of BMP4 (0, 50, 100, 200, and 500 ng/mL) on germ cell and gonadal somatic cell differentiation (Figure 6D). NT‐positive cells were absent in the control group without BMP4 (Figure 6D,E). The proportion of NANOS3/TFAP2C/NANOG triple‐positive cells increased in a concentration‐dependent manner, peaking at 44.9% with 500 ng/mL BMP4 (Figure 6E,F). Furthermore, BMP4 not only activated NANOS3 but also promoted DDX4 expression, a late PGC marker; however, the yield of NANOS3/NANOG/DDX4 triple‐positive cells remained low, reaching only 11.8% at 500 ng/mL BMP4 (Figure 6G,H). Consistent with findings in mice and humans, BMP signaling plays a conserved role in directing early PGCs toward oogonia and pro‐spermatogonia across species. In addition, BMP4 also promoted gonadal somatic cell differentiation in a dose‐dependent manner (Figure 6I). The proportion of SOX9^+^GATA4^+^ cells progressively increased to 8.7% at 500 ng/mL BMP4, while KRT19^+^GATA4^+^ cells showed a similar trend, reaching 21.3% at the same concentration (Figure S9E–G). Together, BMP4 activates germline markers, including NANOS3 and DDX4, and also promotes the differentiation of gonadal somatic cells, as indicated by increased GATA4 co‐expression with SOX9 or KRT19.

**FIGURE 6:**
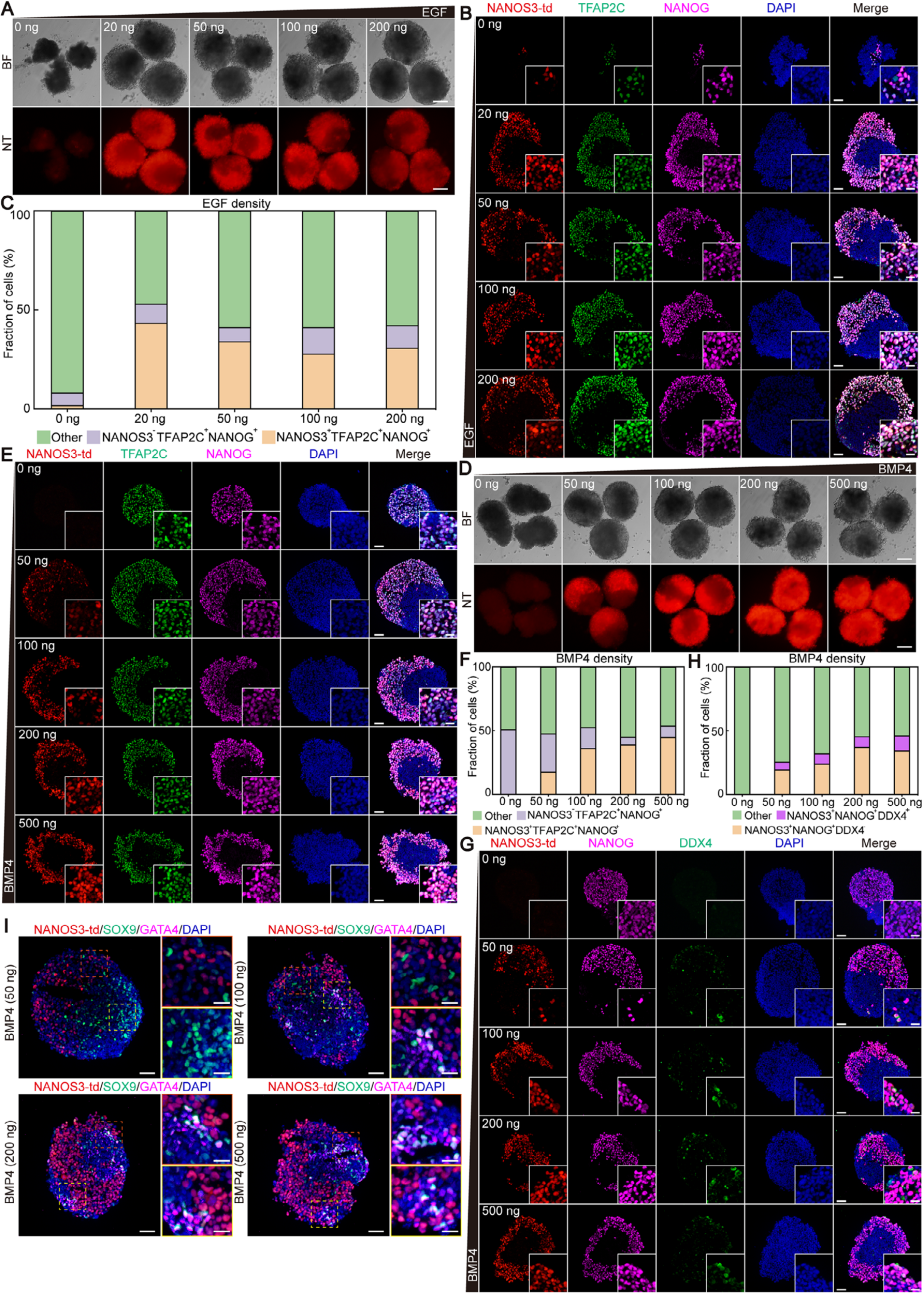
Dose‐dependent effects of EGF and BMP4 on germ and somatic cell differentiation. (A) Fluorescence microscope images of NT reporter transgenic male pgEpiSCs cultured for 8 days with induction of SOX17 in the presence of cytokines: BMP4/SCF/LIF/IWR1/Y‐27632 (BSLIY) +EGF doses (0, 20, 50, 100, and 200 ng). Scale bars: 100 µm. (B) IF analyses of the expression of TFAP2C (green) and NANOG (purple) in NT^+^ (red) cells of aggregates induced from transgenic male pgEpiSCs after 8 days induction of SOX17 in the presence of cytokines: BSLIY + EGF doses (0, 20, 50, 100, and 200 ng). The cells were counterstained with DAPI (blue), Scale bars: 50 µm (zoomed‐out), 20 µm (zoomed‐in). (C) Quantification of immunostaining data in Figure 6B. The numbers of Other, NANOS3^−^TFAP2C^+^NANOG^+^ and NANOS3^+^TFAP2C^+^NANOG^+^ induced from transgenic pgEpiSCs after 8 days of induction of SOX17 in the presence of cytokines: BSLIY + EGF doses (0, 20, 50, 100, and 200 ng). (D) Fluorescence microscope images of NT reporter transgenic male pgEpiSCs cultured for 8 days with induction of SOX17 in the presence of cytokines: EGF/SCF/LIF/IWR1/Y‐27632 (ESLIY) + BMP4 doses (0, 50, 100, 200, and 500 ng). Scale bars: 100 µm. (E) IF analyses of the expression of TFAP2C (green) and NANOG (purple) in NT^+^ (red) cells of aggregates induced from transgenic male pgEpiSCs after 8 days induction of SOX17 in the presence of cytokines: ESLIY + BMP4 doses (0, 50, 100, 200, and 500 ng). The cells were counterstained with DAPI (blue), Scale bars: 50 µm (zoomed‐out), 20 µm (zoomed‐in). (F) Quantification of immunostaining data in Figure 6E. The numbers of Other, NANOS3^−^TFAP2C^+^NANOG^+^ and NANOS3^+^TFAP2C^+^NANOG^+^ induced from transgenic pgEpiSCs after 8 of days induction SOX17 in the presence of cytokines: ESLIY + BMP4 doses (0, 50, 100, 200, and 500 ng). (G) IF analyses of the expression of DDX4 (green) and NANOG (purple) in NT^+^ (red) cells of aggregates induced from transgenic in male pgEpiSCs with 8 days induction of SOX17 in the presence of cytokines: ESLIY + BMP4 doses (0, 50, 100, 200, and 500 ng). The cells were counterstained with DAPI (blue), Scale bars: 50 µm (zoomed‐out), 20 µm (zoomed‐in). (H) Quantification of immunostaining data in Figure 6G. The numbers of Other, NANOS3^+^NANOG^+^DDX4^+^ and NANOS3^+^NANOG^+^DDX4^−^ induced from transgenic pgEpiSCs with 8 days induction of SOX17 in the presence of cytokines: ESLIY +BMP4 doses (0, 50, 100, 200, and 500 ng). (I) IF analyses of the expression of SOX9 (green) and GATA4 (purple) in NT^+^ (red) cells of aggregates induced from transgenic in male pgEpiSCs with 8 days induction of SOX17 in the presence of cytokines: ESLIY + BMP4 doses (50, 100, 200, and 500 ng). The cells were counterstained with DAPI (blue), Scale bars: 50 µm (zoomed‐out), 20 µm (zoomed‐in).

### 2.7 EGF is Required for pPGCLC Survival in xrOvaries

Although mouse fetal gonadal somatic cells are readily available, cost‐effective, and widely used in xrOvaries and xrTestes to support hPGCLCs differentiation into oogonia‐like or pro‐spermatogonia‐like cells, they are unable to support the survival of bovine PGCLCs [14, 15, 17]. To investigate whether mouse fetal gonadal somatic cells can support pPGCLCs survival and differentiation, we isolated E13.5 mouse fetal gonadal somatic cells by magnetic cell sorting (MACS) and aggregated them with 8 days NT‐positive cells to generate xrOvaries and xrTestes (Figure 7A) [5, 24]. Given that male germ cells differentiate more efficiently than female germ cells in human xrOvaries, we separately aggregated male and female NT‐positive cells with mouse gonadal somatic cells to generate xrOvaries, thereby avoiding confounding effects of sex differences [14]. Consistent with observations in cattle, NT‑positive cells derived from both male and female pgEpiSCs were not maintained in xrOvaries beyond 8 days (Figure 7B,C) [17]. FACS analysis showed that they had almost completely disappeared by day 8, comprising only 0.19% (male) and 0.25% (female) of total cells (Figure 7D). These results suggest that mouse fetal gonadal somatic cells have limited capacity to support large‑animal germ cells due to species‑specific differences.

**FIGURE 7:**
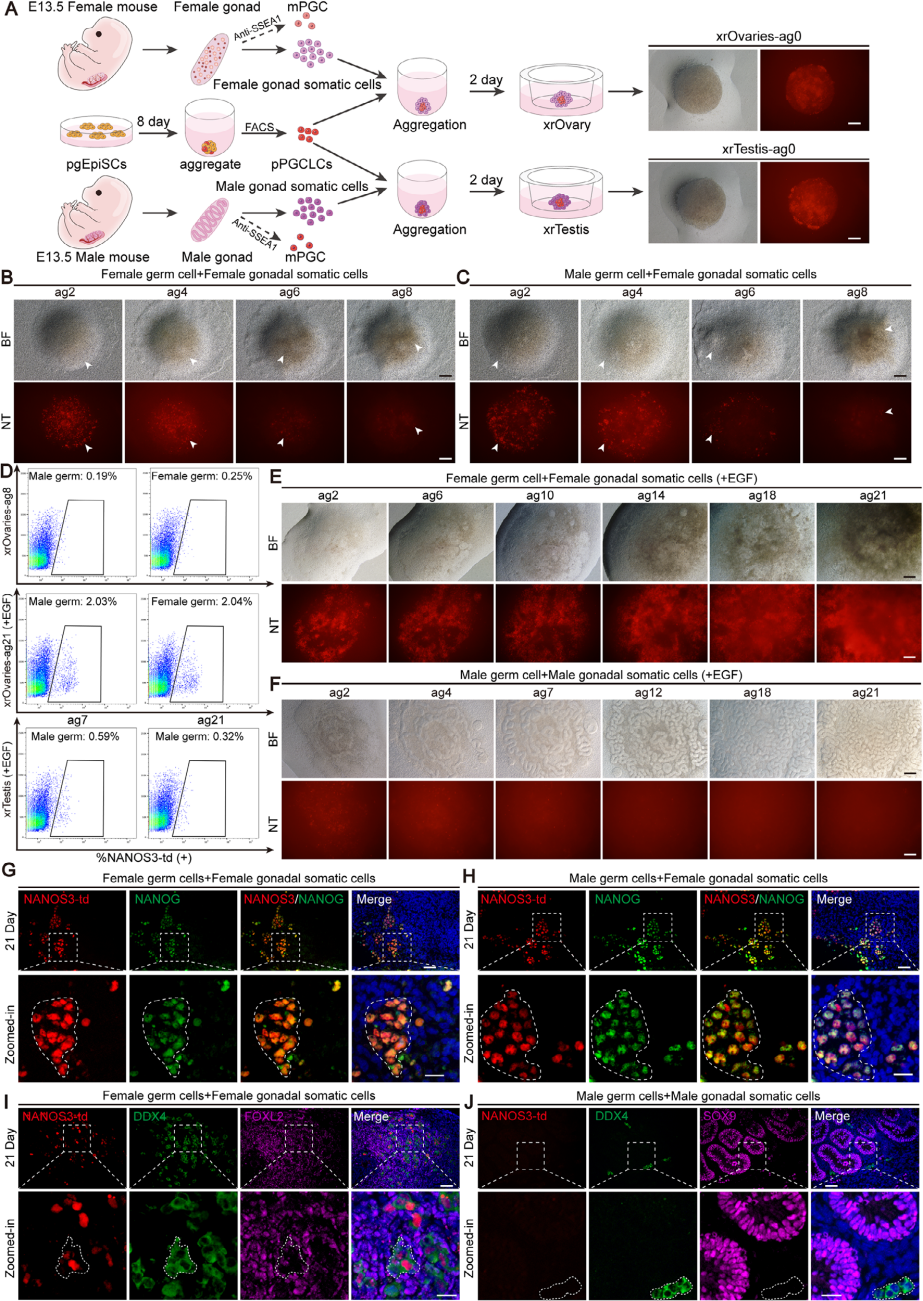
Culture and Analysis of xrOvaries and xrTestes. (A) Schematic diagram of xrOvaries and xrTestes, which were cultured in suspension for two days and then transferred to Transwell‐COL, at which point they were designated as ag0. Scale bars: 200 µm. (B) Fluorescence microscopy images of xrOvaries. NT‐positive cells, derived from female pgEpiSCs following induction with SOX17/TFAP2C for 8 days, were aggregated with mouse E13.5 gonadal somatic cells. The aggregates were cultured in aRB27 medium. Scale bars: 200 µm. (C) Fluorescence microscopy images of xrOvaries. NANOS3‐tdTomato positive cells, derived from male pgEpiSCs following induction with SOX17 for 8 days, were aggregated with mouse E13.5 gonadal somatic cells. The aggregates were cultured in aRB27 medium. Scale bars: 200 µm. (D) Flow cytometry of NT^+^ cells in xrOvaries and xrTestes: xrOvaries (top) cultured in aRB27 basal medium for 8 days (male left, female right); xrOvaries (middle) with EGF for 21 days (male left, female right); xrTestes (bottom) with EGF (7days left, 21days right). (E) Fluorescence microscopy images of xrOvaries. NT‐positive cells, derived from female pgEpiSCs following induction with SOX17/TFAP2C for 8 days, were aggregated with mouse E13.5 gonadal somatic cells. The aggregates were cultured in aRB27 basal medium supplemented with EGF for 21 days. Scale bars: 200 µm. (F) Fluorescence microscopy images of xrTestes. NT‐positive cells, derived from male pgEpiSCs following induction with SOX17 for 8 days, were aggregated with mouse E13.5 gonadal somatic cells. The aggregates were cultured in aRB27 basal medium supplemented with EGF for 21 days. Scale bars: 200 µm. (G) IF analyses of xrOvaries at day 21 of culture showing NT^+^ (red, female) and NANOG (green) expression. The cells were counterstained with DAPI (blue), and merged images are shown on the right. Scale bars: 50 µm (zoomed‐out), 20 µm (zoomed‐in). (H) IF analyses of xrOvaries at day 21 of culture showing NT^+^ (red, male) and NANOG (green) expression. The cells were counterstained with DAPI (blue), and merged images are shown on the right. Scale bars: 50 µm (zoomed‐out), 20 µm (zoomed‐in). (I) IF analyses of xrOvaries at day 21 of culture showing NT^+^ (red, female), DDX4 (green), and FOXL2 (purple) expression. The cells were counterstained with DAPI (blue), and merged images are shown on the right. Scale bars: 50 µm (zoomed‐out), 20 µm (zoomed‐in). (J) IF analyses of xrTestes at day 21 of culture showing DDX4 (green) and SOX9 (purple) expression. The cells were counterstained with DAPI (blue), and merged images are shown on the right. Scale bars: 50 µm (zoomed‐out), 20 µm (zoomed‐in).

Given the indispensable role of EGF in pPGCLCs differentiation, we speculated that EGF might also be critical for pPGCLCs survival. We therefore added EGF to the xrOvaries culture medium. Under this condition, both male‐ and female‐derived NT‐positive cells survived and expanded, with FACS showing proportions of 2.03% and 2.04%, respectively, and numerous NT‐positive cells still detectable in the outer region after 21 days (Figure 7D,E and Figure S10A). In the xrTestes system, however, pPGCLCs failed to survive even with EGF supplementation, with FACS showing NT‐positive cell proportions of 0.59% at day 7 and 0.32% at day 21 (Figure 7D,F). Immunofluorescence staining was performed to examine the distribution of pPGCLCs within xrOvaries and xrTestes. In xrOvaries, NT‐positive cells co‐expressed the pluripotency marker NANOG as well as the late PGC markers DDX4 and DAZL (Figure 7G,H and Figure S10B,C). These NT‐positive cells localized to the outer region of the tissue and were surrounded by FOXL2‐positive mouse granulosa cells (Figure 7I and Figure S10D). This spatial distribution resembles the cortical localization pattern observed in porcine and human female germ cells during early gonad development [30, 49]. In contrast, only a few clustered cells expressing DDX4 were detected in xrTestes; these cells failed to integrate effectively into the mouse seminiferous tubule structures and were primarily distributed outside the tubules (Figure 7J and Figure S10E). This abnormal distribution may explain the inability of pPGCLCs to survive and expand in xrTestes. In summary, EGF supplementation in the xrOvaries culture medium not only supported pPGCLCs survival but also resulted in a spatial distribution pattern that resembles that of early porcine gonads.

### 2.8 Establishment of a Long‐Term Expandable Culture System for pPGCLCs

Although the one‐step system efficiently generates oogonia‐like and pro‐spermatogonia‐like cells, the limited cell yield hinders mechanistic dissection of germ cell expansion and restricts potential downstream applications such as stem cell‐based breeding. We therefore developed a long‐term expansion module to overcome this bottleneck. Long‐term in vitro expansion of PGCs has been established in mice and humans but not in large animals such as pigs [19, 54, 58]. To explore whether existing protocols could be adapted, we first tested whether mouse and human PGCLCs expansion media could support long‐term in vitro expansion of pPGCLCs (Figure 8A). The mouse medium consisted of GK15 + 2.5% FBS supplemented with Forskolin, Rolipram and SCF, while the human medium consisted of GK15 + 2.5% FBS supplemented with Forskolin, FGF2 and SCF. We isolated NT‐positive pPGCLCs by FACS on day 8 and plated them on SNL feeders for expansion (Figure 8A). The number of NT‐positive cells began to decrease after day 4 and was barely detectable by day 10 (Figure S11A–C). Although NT‐positive cells retained pPGCLCs molecular characteristics at day 4, expression of key pluripotency and PGC markers was barely detectable by day 10 (Figure S11D–F). These results suggest that mouse and human PGCLCs expansion media fail to support sustained expansion of pPGCLCs beyond 10 days.

**FIGURE 8:**
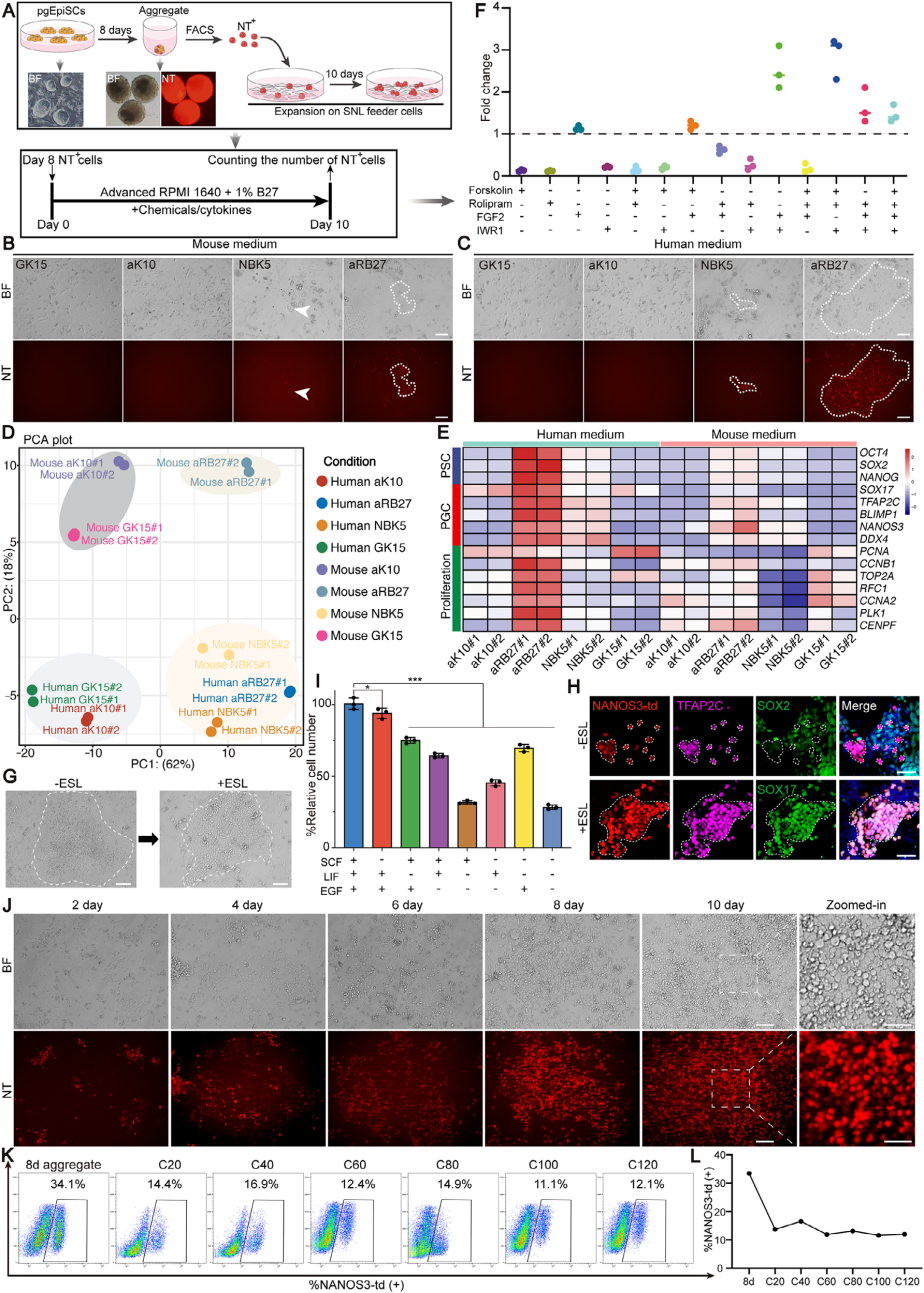
Establishment of an in vitro long‐term expansion culture system for pPGCLCs. (A) Experimental scheme for extended culture of pPGCLCs on SNL feeder cells (Top). Day 8 pPGCLCs were sorted by FACS as NT‐positive cells. A scheme for the exploration of a condition for NT^+^ cell expansion (Bottom). (B) Representative fluorescence microscopy images of NT‐positive pPGCLCs sorted on day 8 and expanded for 10 days in different basal media (GK15, aK10, NBK5, and aRB27) under mouse medium (Forskolin/Rolipram/SCF). Scale bars: 100 µm. (C) Representative fluorescence microscopy images of NT‐positive pPGCLCs sorted on day 8 and expanded for 10 days in different basal media (GK15, aK10, NBK5, aRB27) under human medium (Forskolin/FGF2/SCF). Scale bars: 100 µm. (D) PCA plot of pPGCLCs expanded for 10 days under various culture conditions. The transcriptomic clustering of pPGCLCs cultured in four basal media (GK5, aK10, NBK5, and aRB27) using mouse and human long‐term expansion culture media for 10 days is shown. (E) Heatmap of lineage‐specific gene expression in pPGCLCs cultured for 10 days in different conditions. Sample ordering: culture system (human/mouse medium) and basal medium (GK15, aK10, NBK5, aRB27). Gene clustering: PSC, PGC, and proliferation markers. Color scale: relative expression levels. (F) A plot showing the number of NT^+^ cells after 10 days of culture under the indicated conditions, as determined by FACS analysis. The factors tested included Forskolin, Rolipram, IWR1, and FGF2. (G) Fluorescence microscopy images of NT‐positive pPGCLCs expanded for 20 days. Left: culture medium with aRB27 + FGF2/Forskolin/IWR1. Right: culture medium with aRB27 + FGF2/Forskolin/IWR1 + EGF/SCF/LIF. Scale bars: 100 µm. (H) IF images showing marker expression in pPGCLCs after 20 days of expansion. ‐ESL condition (top): NT^+^ (red), SOX2 (green), TFAP2C (purple). + ESL condition (bottom): NT^+^ (red), SOX17 (green), TFAP2C (purple). DAPI (blue) counterstaining. Scale bars: 50 µm. (I) Relative numbers of long–term pPGCLCs expanded in a 10‐day culture in the presence of indicated growth factors (EGF, SCF, and LIF, ESL). (mean ± SEM). Cell numbers of the condition containing ESL are defined as 100%; ns, *P* > 0.05, ^*^P < 0.05, ^**^P < 0.01, ^***^P < 0.001. Error bars represent mean ± SEM. (J) Representative fluorescence microscopy images showing the expansion process of NT^+^ cells from day 2 to day 10. The dashed box indicates the magnified area showing pPGCLCs at day 10. Scale bars: 100 µm (zoomed‐out), 50 µm (zoomed‐in). (K) FACS plots for NT^+^ expression at day 8 of pPGCLC induction and at passages 10–120 (with FACS purification every 20 days) of NT^+^ cell expansion culture starting from day 8 pPGCLCs on SNL feeders in aRB27 + FGF2/Forskolin/IWR1/EGF/SCF/LIF. (L) The transitions of the percentages of NT^+^ cells during the pPGCLCs expansion culture until C120.

Due to the distinct basal media used for PGCLCs induction in mice and humans vs. pigs, we hypothesized that the type of basal medium critically influences long‐term expansion [9, 24]. To test this, we designed four basal media—GK15 (GMEM + 15% KOSR), aK10 (advanced RPMI 1640 + 10% KOSR), NBK5 (N2/B27 + 5% KOSR), and aRB27 (advanced RPMI 1640 + 1% B27)—and combined each with either mouse (Forskolin, Rolipram and SCF) or human (Forskolin, FGF2 and SCF) expansion supplements. Interestingly, in KOSR‐containing media (GK15, aK10), pPGCLCs failed to survive regardless of supplement (Figure 8B,C). In contrast, in B27‐containing media (NBK5, aRB27), NT‐positive cells were maintained until day 10 without expansion, with the human formulation performing markedly better than the mouse formulation. To assess transcriptional effects, we cultured pPGCLCs for 10 days in four basal media with mouse or human formulations (Figure 8B,C). PCA revealed that expression profiles clustered by basal medium type (KOSR‐containing vs. B27‐containing) (Figure 8D and Figure S12A). Under the human formulation, B27‐containing media, particularly aRB27, showed higher expression of pluripotency, PGC, and proliferation markers, with aRB27 exhibiting the highest levels (Figure 8E). DEG and GSEA further revealed that the human formulation with aRB27 induced enrichment in “steroid metabolic process”, “embryonic development”, and “gamete generation” (Figure S12B–D). Together, these results suggest that the human formulation with aRB27 is most suitable for maintaining pPGCLCs survival.

Given that Forskolin and Rolipram support mPGCLCs survival via cAMP activation, FGF2 is essential for hPGCLCs survival, and IWR1 enhances pPGCLCs differentiation through WNT inhibition, we selected these four factors for combinatorial screening using aRB27 as the basal medium to establish a long‐term expansion system for pPGCLCs (Figure 8A,F) [58]. After 10 days of culture, we found that the combination of FGF2 and IWR1 supported pPGCLCs expansion; addition of Forskolin further enhanced expansion efficiency, whereas Rolipram appeared to impair expansion (Figure 8F). We therefore proceeded with the medium containing FGF2, IWR1, and Forskolin for further optimization. When cultured in this medium for 20 days, the grape‐like pPGCLCs transitioned to an epithelial‐like morphology, with a subset of cells differentiating into SOX2‐positive embryonic germ cell (EGC)‐like cells (Figure 8G,H) [59]. To suppress this unwanted differentiation, we supplemented EGF, which we previously found to support pPGCLCs survival in xrOvaries, into the medium. EGF significantly improved pPGCLC expansion efficiency (Figure 8G–I). Furthermore, adding SCF and LIF further reduced the number of EGC‐like cells while markedly increasing the proportion of grape‐like pPGCLC clusters (Figure 8I).

Given the role of BMP2 in human pro‐spermatogonia and oogonia differentiation, we next investigated BMP2, BMP4, and BMP7 in pPGCLCs expansion culture (Figure S13A) [60]. PCA showed that BMP4‐treated samples formed a distinct cluster from BMP2/7‐treated samples. BMP4 significantly upregulated the germ cell marker DDX4 and the BMP target genes *ID2* and *ID3*, with GSEA revealing enrichment in “stem cell development” and “sex differentiation” (Figure S13B–D and Table S6). However, immunofluorescence detected DDX4‐positive cells regardless of exogenous BMP supplementation, indicating that BMP4 may induce late PGC features but is not required for maintenance; supplementation with BMP2, BMP4, or BMP7 consistently reduced the proliferation capacity of pPGCLCs, in contrast to the indispensable role of BMP2 in humans (Figure S13E,F). We next used the established expansion system (aRB27 basal medium supplemented with FGF2, Forskolin, IWR1, EGF, SCF, and LIF) for long‐term expansion of pPGCLCs, without exogenous transcription factor activation. Under these conditions, NT‐positive cells expanded stably and were passaged every 10 days (Figure 8J). To ensure the purity of NT‐positive cells, FACS was performed every 20 days, and NT‐positive cells were successfully maintained in culture for up to 120 days (C120) (Figure 8K,L). This culture system supported the long‐term in vitro expansion of both female and male pPGCLCs, although female pPGCLCs proliferated at a notably slower rate, approximately half that of their male counterparts (Figure S13G,H). In summary, we established an expansion system for pPGCLCs based on aRB27 supplemented with FGF2, IWR1, Forskolin, EGF, LIF, and SCF.

### 2.9 Long‐Term Expanded pPGCLCs Maintain PGC and Pro‐Spermatogonia Characteristics

To further investigate the molecular characteristics of pPGCLCs during expansion culture, we collected samples at days C10, C30, C60, and C120 for qPCR analysis (Figure S14A). The results showed that NT‐positive cells in long‐term expansion expressed pluripotency genes *OCT4* and *NANOG* at levels similar to pgEpiSCs, downregulated *SOX2*, and maintained high expression of pPGC markers *TFAP2C*, *BLIMP1*, and *NANOS3* throughout the culture period (Figure S14A). Of note, NT‐positive cells in long‐term expansion exhibited significantly reduced *SOX17* expression relative to day 8 differentiated cells, consistent with the absence of exogenous SOX17 activation (Figure S14A). Additionally, we observed that *DAZL* and *DDX4* were induced during expansion (Figure S14A). Immunofluorescence analysis at days 10 and 30 showed that NT‐positive cells co‐expressed early PGC markers OCT4, NANOG, TFAP2C, and BLIMP1, confirming sustained early PGC molecular characteristics under this expansion system (Figure 9A). We further examined the expression of oogonia and pro‐spermatogonia markers DAZL and DDX4 at days 10, 30, and 60 of culture (Figure 9B). The results showed that only a subset of NT‐positive cells expressed DAZL and DDX4, indicating the coexistence of cells at different differentiation states within this expansion system. Together, these results indicate that this expansion system maintains the molecular characteristics of early PGCs while also supporting the emergence of pro‐spermatogonia‐like cells, without requiring exogenous transcription factor activation.

**FIGURE 9:**
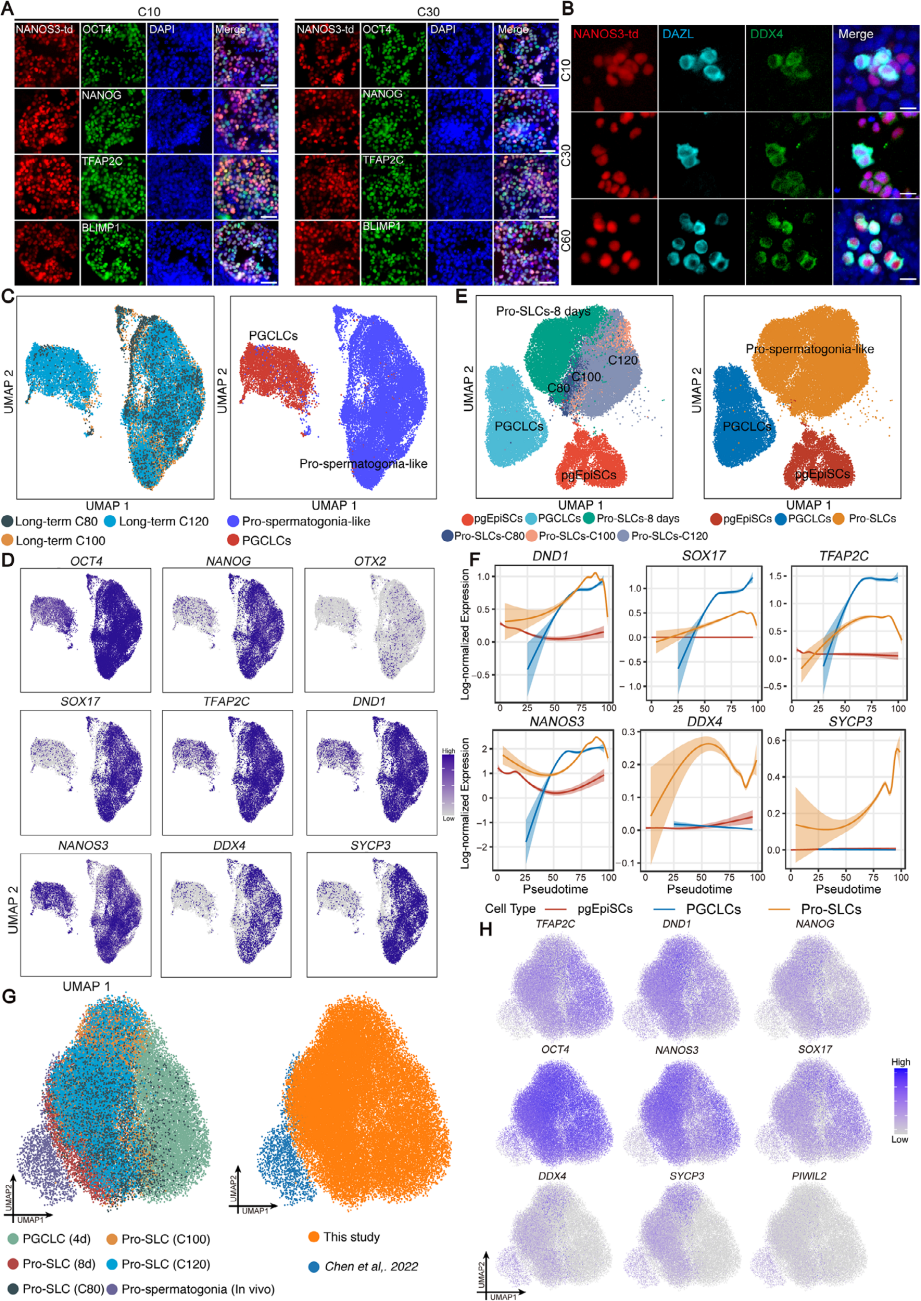
Molecular characterization of oogonia‐like and pro‐spermatogonia‐like cells in the long‐term expansion system. (A) IF analyses of the expression of OCT4, NANOG, TFAP2C, and BLIMP1 (green) in NT^+^ (red) cells during NT^+^ cell expansion culture at C10 (left) and at C30 (right). The cells were counterstained with DAPI (blue). Scale bars: 50 µm. (B) IF analyses of the expression of DAZL (cyan) and DDX4 (green) in NT^+^ (red) cells during NT^+^ cell expansion culture at C10 (Top), C30 (middle), and C60 (bottom). Scale bars: 20 µm. (C) UMAP visualization of cell populations derived from male pPGCLCs cultured in the long‐term expansion system for 80, 100, and 120 days. (D) UMAP visualization of representative genes germ cells. Expression levels were color coded. (E) UMAP visualization of male pgEpiSCs, d4 pPGCLCs, d8 pro‐spermatogonia‐like cells, and pro‐spermatogonia‐like cells expanded for 80, 100, and 120 days in the long‐term expansion system. (F) Pseudotime‐ordered gene expression dynamics for key germ cell and pluripotency‐related genes across three cell populations: pgEpiSCs, PGCLCs, and pro‐spermatogonia‐like (ProSLCs). The x‐axis represents pseudotime, and the y‐axis represents log‐normalized gene expression levels. (G) UMAP visualization of integrated cell populations including male pgEpiSC‐derived PGCLCs, pro‐spermatogonia‐like cells (day 8 of differentiation, and long‐term expanded for 80, 100, and 120 days), and porcine male fetal germ cells (pro‐spermatogonia). Cells are colored by annotated cell types. (H) UMAP visualization of representative genes for male PGCLCs, Pro‐spermatogonia‐like cells, and porcine fetal germ cells (Pro‐spermatogonia). Expression levels were color–coded.

To further investigate the transcriptional landscape underlying this maintenance, we performed single‐cell RNA sequencing on male NT‐positive cells cultured for 80 (C80), 100 (C100), and 120 days (C120) (Figure 9C). Throughout the long‐term expansion process, only two cell types were identified—pPGCLCs and pro‐spermatogonia‐like cells—consistent with our immunofluorescence staining observations (Figure 9D,E). Both germ cell types were detected at days 80, 100, and 120, with a consistently higher proportion of pro‐spermatogonia‐like cells across all time points, suggesting that both germ cell states are stably maintained during long‐term expansion (Figure 9C,D and Figure S14B). Single‐cell transcriptomics resolved two distinct germ cell states: pPGCLCs, characterized by high expression of early PGC markers (*BLIMP1*, *TFAP2C*, *NANOS3*) and absence of late PGC markers, and pro‐spermatogonia‐like cells, characterized by co‐expression of both early and late PGC markers (*DDX4*, *SYCP3*) (Figure 9C,D). Collectively, these results indicate that the long‐term expansion system sustains the distinct molecular characteristics of both pPGCLCs and pro‐spermatogonia‐like cells across different time points.

We next asked whether the pro‐spermatogonia‐like cells maintained in the expansion system resemble those derived at day 8 of differentiation. To address this, we performed an integrated analysis of pgEpiSCs, pPGCLCs, day 8 differentiated pro‐spermatogonia‐like cells, and pro‐spermatogonia‐like cells from various long‐term culture time points (Figure 9E). Day 8 differentiated pro‐spermatogonia‐like cells clustered together with pro‐spermatogonia‐like cells from different culture durations into a single major cluster, indicating transcriptional similarity (Figure 9E). Notably, pseudotime analysis revealed that pro‐spermatogonia‐like cells under long‐term culture conditions were positioned at the terminal end of the developmental trajectory, suggesting they may represent a later stage of pro‐spermatogonia (Figure S14C). PGC markers (*DND1*, *SOX17*, and *TFAP2C*) were minimally expressed in pgEpiSCs but progressively increased in pPGCLCs and pro‐spermatogonia‐like cells, whereas *DDX4* and *SYCP3* were upregulated specifically during pro‐spermatogonia‐like differentiation (Figure 8F and Figure S14D). Transcriptional profiling further confirmed that the long‐term expansion system maintained both early and late PGC markers, while pluripotency genes (*SOX2*, *OTX2*) remained barely detectable in germ cells (Figure S14E). At the epigenetic regulator level, long‐term cultured pro‐spermatogonia‐like cells displayed low *DNMT3B* and high *TET1*, *POLB*, and *PARP1* expression relative to pgEpiSCs, suggesting that these cells express epigenetic regulators consistent with germ‐cell‐like epigenetic remodeling. Histone modification genes (*RBBP7*, *DPY30*, *HLTF*, *SMARCA5*) were also expressed at levels comparable to day 8 differentiated cells (Figure S14F) [22].

To relate these in vitro populations to their in vivo counterparts, we next compared the differentiated PGCLCs and pro‑spermatogonia‑like cells, as well as the long‑term cultured pro‑spermatogonia‑like cells from different time points, with porcine fetal pro‑spermatogonia [23]. Integrative analysis showed that day 8 pro‑spermatogonia‑like cells clustered closely with the long‑term cultured pro‑spermatogonia‑like cells, and all of these populations expressed in vivo pro‑spermatogonia markers such as *DDX4*, *SYCP3*, and *PIWIL2* (Figure 9G,H). In addition, these pro‑spermatogonia‑like cells highly expressed early PGC markers including *TFAP2C*, *NANOG*, and *NANOS3* (Figure 9G,H). Thus, long‑term expanded pro‑spermatogonia‑like cells retain the molecular characteristics of pro‑spermatogonia and are transcriptionally aligned with in vivo porcine fetal pro‑spermatogonia. In summary, these results indicate that pro‑spermatogonia‑like cells derived from pgEpiSCs maintain their molecular characteristics for up to 120 days in the expansion system and are transcriptionally aligned with in vivo porcine fetal pro‑spermatogonia.

## 3 Discussion

Mice have advanced gametogenesis research due to their short reproductive cycle and low cost, while differing from humans in developmental timing and germ cell regulation [61]. Pigs, with embryonic morphology, organ size, and germ cell regulatory networks resembling humans, offer an optimal model [62]. Moreover, as a large animal species, the pig enables experimental manipulations—such as germline gene editing and large‐scale gonadal tissue analysis—that are ethically or practically infeasible in humans, and offers a unique platform for developing stem cell‐based breeding strategies. However, directed differentiation of porcine PSCs into germ cells remains underdeveloped, with neither pro‐spermatogonia/oogonia induction nor an in vitro expansion system established, limiting their application in stem cell‐based breeding and regenerative medicine [10, 24]. In this study, we established a modular platform for the direct differentiation and long‐term expansion of porcine germ cell‐like cells. We first developed a one‐step method to directly differentiate pgEpiSCs into pro‐spermatogonia‐like cells (via SOX17 activation in males) and oogonia‐like cells (via SOX17/TFAP2C co‐activation in females), with concomitant induction of gonadal somatic‐like cells. Mechanistically, we found that EGF activates the core PGC regulatory network. In xrOvaries, EGF also sustained cell survival and reproduced the cortical distribution characteristic of early gonadal development. The combination of BMP4 and EGF was essential for activating NANOS3, inducing the late germ cell markers DAZL and DDX4, as well as promoting GATA4 expression in somatic cells. Finally, we established an exogenous transcription factor‑independent expansion system that maintained pPGCLCs with early PGC and pro‐spermatogonia characteristics for up to 120 days, providing a foundation that may support future downstream applications such as stem cell‐based breeding.

Species‐specific differences in pluripotency states lead to divergent culture conditions and distinct PGCLCs induction strategies. In mice, naive mESCs cannot directly respond to BMP4 and require a transition through EpiLCs, whereas primed epiblast stem cells (EpiSCs) lack this competence [24]. Recently established formative PSCs in mice and humans can directly respond to BMP4 for efficient PGCLC induction [63, 64, 65]. In contrast, primed hiPSCs are first induced into iMeLCs/Pre‐Me for efficient hPGCLCs differentiation [9, 10]. In both mice and humans, the core PGC regulatory network cannot be directly activated at the pluripotent stage; cells must first acquire a competent state. In mEpiLCs, PRDM14 alone initiates the network, while co‐activation with BLIMP1 and TFAP2C enables cytokine‐free induction [13, 66]. In human iMeLCs, SOX17, TFAP2C, and BLIMP1 still require BMP4, whereas combining GATA2/GATA3 with SOX17/TFAP2C achieves cytokine‐free hPGCLCs induction (Table 1) [67]. These findings highlight species‐specific mechanisms in PGCLCs induction. In this study, pgEpiSCs derived from porcine E10 epiblast express the formative marker *OTX2* and exhibit a formative‐like transcriptome, yet show limited chimeric contribution and fail to respond to BMP4 for pPGCLC differentiation [20]. By contrast, E11 epiblast cells can form pPGCLCs upon cytokine induction, suggesting that E11 epiblast cells may represent a true formative state while E10 corresponds to an earlier pluripotent state [10]. This functional divergence may explain why pgEpiSCs cannot be directly induced into pPGCLCs, yet retain the potential to differentiate through exogenous transcription factor activation. Moreover, male pgEpiSCs require only SOX17 activation to induce pro‐spermatogonia‐like cells, whereas female pgEpiSCs need SOX17/TFAP2C co‐activation for oogonia‐like cell formation, likely due to partial X chromosome inactivation in females, a trend consistent with the generally weaker differentiation capacity of female human PSCs [68]. Beyond this, we observed that male pgEpiSCs expressed slightly higher levels of pluripotency markers *OCT4*, *SOX2*, and *NANOG*, whereas female cells exhibited modestly elevated *OTX2* expression. Given that elevated *OTX2* levels have been reported to suppress PGCLC differentiation efficiency—while its depletion enhances it—the modestly elevated *OTX2* expression in female cells may contribute to their reduced induction efficiency. During long‐term expansion, oogonia‐like cells also proliferated substantially slower than pro‐spermatogonia‐like cells. This may reflect the intrinsic developmental programs of the two sexes: female germ cells directly enter meiosis after sex determination, whereas male germ cells undergo a dedicated mitotic expansion phase before entering mitotic arrest, making the proliferative window of oogonia fundamentally narrower than that of pro‐spermatogonia.

**TABLE 1:** Cross‐species comparison of PGCLC derivation and expansion characteristics.

Feature	Mouse	Human	Porcine (This study)
Starting pluripotent stem cells	mESCs	hPSCs/hiPSCs	pgEpiSCs
Intermediate step before PGCLC induction	mEpiLCs (Activin A + bFGF)	iMeLCs (Activin A + CHIR)	—
Chemical induction for PGCLC	Effective on EpiLCs	Effective on iMeLCs	Not effective on pgEpiSCs
TF‐mediated PGCLC induction	PRDM14 (in EpiLCs)	SOX17 (in iMeLCs)	Male: SOX17 Female: SOX17 + TFAP2C
Key growth factor requirement	BMP4	BMP4	BMP4 + EGF
WNT inhibition response	IWR1 enhances efficiency	IWR1 enhances efficiency	IWR1 enhances efficiency
Core PGC regulatory network	BLIMP1, TFAP2C, PRDM14	SOX17, TFAP2C, BLIMP1	SOX17, TFAP2C, BLIMP1
SOX2 repression	Maintained	Repressed	Repressed
Direct generation of late PGCLCs	No	No	Yes
Co‐differentiation of germ and gonadal somatic cells	Not reported	Not reported	Yes
Co‐culture with mouse gonadal somatic cells	rOvary/rTestis supportive	xrOvary/xrTestis supportive	xrOvary supportive (EGF‑dependent); xrTestis not supportive
Long‑term expansion medium	Forskolin + Rolipram + SCF	Forskolin + FGF2 + SCF	FGF2 + Forskolin + IWR1+ EGF + SCF + LIF

Although the core pluripotency network is highly conserved across species, as evidenced by OCT4, SOX2, and NANOG expression, culture conditions for PSCs show marked species specificity. The 2i/LIF medium that supports mESCs fails to sustain PSCs from large animals, with particular challenges in exogenous gene silencing during iPSC generation. Recent single‐cell transcriptomic analyses of early pig embryogenesis have revealed that WNT pathway inhibitors, especially IWR1, are essential for establishing large animal PSCs and ensuring efficient exogenous gene silencing in porcine iPSCs [20, 28, 69, 70, 71]. Notably, IWR1 plays multiple roles in germ cell development: it maintains pgEpiSCs, enhances pPGCLCs induction efficiency by suppressing mesodermal fate, and is essential for pPGCLCs expansion. This mechanism is consistent with the promotive effect of IWR1 in hPGCLCs differentiation, suggesting conserved function across mammals (Table 1) [29]. Collectively, these findings highlight IWR1 as a key regulator across pluripotency maintenance, germline induction, and expansion, providing insights into pluripotency and germline commitment in porcine and other large animal species.

Gonadal somatic cells originate from the intermediate mesoderm, giving rise to genital ridge precursors that commit to Sertoli or granulosa cells under sex‐determination signals [52, 72]. These cells are critical for germ cell differentiation and gamete formation. However, current in vitro systems rarely achieve simultaneous germ cell and gonadal somatic cell differentiation. During the differentiation of hiPSCs into hPGCLCs, expression of late PGC markers such as *DAZL* and *DDX4* remains undetectable even after prolonged culture (up to day 12) (Table 1) [9]. In contrast, aggregating mouse or human PGCLCs with mouse gonadal somatic cells significantly upregulates these markers, underscoring the essential role of gonadal somatic cells in promoting PGCs maturation [15, 24]. In our system, gonadal somatic cells spontaneously co‐differentiated alongside pPGCLCs and promoted their further development into pro‐spermatogonia‐like or oogonia‐like cells. We hypothesize that this co‐differentiation phenomenon is closely associated with the unique developmental state of porcine epiblast stem cells and the signaling characteristics of the in vitro differentiation microenvironment. As described above, pgEpiSCs from E10 embryos exhibit a formative‐like state but are not directly competent for PGCLC induction, while E11 epiblast represents a more advanced formative state permissive for germline induction [10, 20]. This distinct developmental status may endow pgEpiSCs with the potential for bidirectional differentiation into both germ cells and gonadal somatic cells under appropriate conditions. With respect to the signaling environment, BMP4 and EGF are indispensable cytokines for germ cell induction in our system, and these same factors are also key regulators of gonadal somatic cell differentiation in mice [73, 74]. This overlap of factor requirements suggests that a common set of signals may concurrently activate both differentiation programs. Notably, in mouse pluripotent stem cell‐derived somatic cell cultures, gonadal somatic cells are predominantly localized to the outer regions of aggregates, whereas in our system, the gonadal somatic‐like cells are more frequently distributed within the aggregate interior. This spatial difference may be attributable to a BMP4 concentration gradient effect: the high BMP4 concentration at the aggregate surface favors pPGCLC specification, while the progressively lower BMP4 concentration toward the interior may approach conditions more suitable for gonadal somatic cell differentiation, thereby spontaneously giving rise to granulosa‐like and Sertoli‐like cells. From an in vivo developmental perspective, during early gonadal specification, germ cells and gonadal somatic cells are spatially juxtaposed and establish a mutually supportive microenvironment, with each population promoting the fate determination and differentiation of the other through paracrine signals. The co‐emergence of germ cells and somatic‐like cells in our in vitro induction system may therefore represent a recapitulation of this in vivo cooperative developmental process, reflecting an intrinsic coupling of germ cell and gonadal somatic cell co‐differentiation. This may also explain why, in mouse and human systems, prolonged induction of PGCLCs fails to directly yield oogonia‐like or pro‐spermatogonia‐like cells, as those systems may lack the microenvironmental support provided by concurrently differentiated gonadal somatic cells. However, due to the absence of somatic cell reporters, we could not purify these cells by FACS or aggregate them with pPGCLCs at defined ratios [73, 74]. Therefore, whether such an aggregation system can form seminiferous tubule‐like structures and further promote pPGCLCs differentiation into functional gametes remains to be investigated in future studies.

To validate these identities, we performed integrative analyses with porcine and human fetal germ cell and gonadal somatic cell datasets. The results suggested that both the in vitro‐derived germ cells and the accompanying somatic cells are transcriptionally similar to their in vivo counterparts, providing unbiased external validation and effectively ruling out concerns of circular annotation. However, the oogonia‐like cells still do not activate markers of RA‐responsive oogonia such as *STRA8* and *ZGLP1*, which are prerequisites for meiotic entry [45]. In the male lineage, the expression levels of late germ cell markers in pro‐spermatogonia‐like cells were lower than those in in vivo pro‐spermatogonia. In contrast, male pro‐spermatogonia‐like cells showed only partial global concordance, although selected late markers such as *DDX4* and *SYCP3* followed trends similar to those in vivo. This may reflect a difference in cell state: in vivo pro‐spermatogonia are largely in mitotic arrest, whereas the in vitro cells may not yet have reached this stage. Therefore, promoting further differentiation of oogonia‐like cells toward the meiotic stage and promoting mitotic‐arrest‐associated maturation of pro‐spermatogonia‐like cells remains an important direction for future research.

To further understand the signaling basis of this co‐differentiation, we dissected the distinct roles of BMP4 and EGF. In humans, BMP4 directs PGCLC induction primarily through SOX17 activation [12]. In contrast, in pgEpiSCs, exogenous activation of SOX17 combined with EGF can initiate part of the PGC core regulatory network, inducing expression of TFAP2C, BLIMP1, OCT4, and NANOG, but fails to induce NANOS3 in the absence of BMP4. Although BMP4 alone cannot activate this core network, it is essential for inducing NANOS3 and driving pro‐spermatogonia‐like or oogonia‐like cell differentiation. BMP4 also induces the gonadal somatic cell marker GATA4, suggesting a dual role in promoting co‐differentiation of both germ cells and gonadal somatic cells. Moreover, this dual effect is dose‐dependent, with higher BMP4 concentrations enhancing induction of both lineages, a characteristic also observed in human and mouse systems, suggesting conserved BMP4 signaling in germ cell differentiation across mammals [8, 24].

Gonadal somatic cells from large animals are difficult and costly to obtain. Although mouse gonadal somatic cells support hPGCLCs differentiation, they cannot sustain germ cell survival from large animals such as pigs and cattle, likely due to species‐specific differences in developmental timing [14, 15, 17]. In our previous work, porcine fetal granulosa cells lack FOXL2 expression from E24 to E50, whereas mouse granulosa cells at E12.5 (corresponding to porcine E30–E35) already express FOXL2 widely [30]. This divergence in maturation timing may explain the failure of large‐animal germ cell survival in xrOvaries. We identified EGF as a key factor that supports pPGCLCs survival in xrOvaries and recapitulates spatial features of early porcine gonads, but its effect in xrTestes is distinct. Unlike hPGCLCs, which integrate into mouse seminiferous tubules, pPGCLCs fail to incorporate even with EGF, with only a few cells detected outside the tubules [15, 36]. Thus, further screening for cytokines that promote pPGCLCs integration is needed. Combining mouse gonadal somatic cells as structural support with specific cytokines may offer new strategies for studying gametogenesis.

During early gametogenesis, PGCs are initially scarce but undergo proliferative expansion to ensure sufficient gamete numbers. Establishing an in vitro expansion system for PGCLCs is therefore essential for dissecting the mechanisms governing PGC proliferation, and serves as a key platform for investigating meiotic entry [11]. Furthermore, such a system may provide a novel source of seed cells for future stem cell‐based breeding applications. Expansion culture systems have been established for mouse and human PGCLCs [19, 58, 75]. In this study, we developed a stable, exogenous transcription factor‑independent expansion system for pPGCLCs. Similar to PSC culture, PGCLC expansion exhibits species specificity. In mice, long‐term expansion of mPGCLCs is achieved simply with Forskolin and Rolipram, but this condition does not apply to pPGCLCs [58]. By contrast, pPGCLCs culture conditions more closely resemble those for human hPGCLCs, as both require FGF2 signaling, though pPGCLCs additionally depend on IWR1 and EGF [60]. Furthermore, the basal culture medium is critical for long‐term maintenance of pPGCLCs, as KOSR fails to support their survival. In humans, BMP2 induces PGCLC differentiation into pro‐spermatogonia‐like and oogonia‐like cells without somatic cell support [60]. In our system, pPGCLCs maintained these characteristics during long‐term culture without additional BMP factors, likely due to prior BMP signaling activation during differentiation. When BMPs were added, pPGCLCs responded better to BMP4 than to BMP2 or BMP7, highlighting species‐specific signaling requirements. Importantly, long‐term maintenance in this system was achieved under periodic FACS purification of NANOS3‐tdTomato‐positive cells, indicating that the culture supports sustained germ cell expansion under repeated reporter‐positive selection rather than autonomous stability of the unsorted culture. In summary, our work has established an in vitro system that supports both the differentiation and long‐term expansion of pPGCLCs, with single‐cell validation of male pro‐spermatogonia‐like cells and marker‐level support for female oogonia‐like cells for up to 120 days. This system offers a valuable model for deciphering porcine gametogenesis and may provide insights for in vitro gamete studies in humans and other large mammals.

### 3.1 Limitations of the Study

Although the induced and long‐term expanded pro‐spermatogonia‐like and oogonia‐like cells resemble their in vivo counterparts at the transcriptional level, functional competence remains to be established. It is not yet determined whether these cells can form functional gametes through transplantation assays, nor whether the co‐differentiated gonadal somatic cells are capable of supporting gametogenesis. In addition, single‐cell transcriptomic profiling of long‐term expanded oogonia‐like cells is currently unavailable. All in vivo comparisons relied on cross‐platform integration, which may introduce technical biases, and cross‐species comparisons with human fetal germ cells were limited to marker‐level analyses without establishing genome‐wide concordance. The in vitro meiotic progression system requires further investigation. Resolving these limitations will be critical for advancing this platform toward functional gametogenesis.

## 4 Materials and Methods

### 4.1 Animals

#### 4.1.1 Ethics Approval

All experimental procedures involving animals were conducted in accordance with the guidelines and regulations for the care and use of laboratory animals and were approved by the Animal Ethics Committee of China Agricultural University (Approval No: AW02113202‐3‐1). We complied with all relevant ethical regulations for animal use.

#### 4.1.2 Mice

The Kunming white mice ((RRID: MGI:5651867)) were obtained from Beijing Sibeifu Biotechnology Co., Ltd. (Beijing, China). Female mice aged 6–8 weeks and male mice aged 8–10 weeks were used for natural mating at a ratio of 2:1. On embryonic day 13.5 (E13.5), fetuses were harvested. Mouse embryonic fibroblasts (MEFs) were isolated for pgEpiSC culture, and fetal gonads were collected for subsequent xenogeneic reconstituted ovary and testis construction. All mice were individually housed under a 12 h light/dark cycle in a sterile environment and provided with food and water ad libitum.

### 4.2 Pig pgEpiSC Culture

All the cells used in this study were maintained in a humidified incubator with 5% CO_2_ at 37°C. Pig pgEpiSCs were cultured as previously described [20]. Briefly, cells were maintained on mitomycin Ctreated mouse embryonic fibroblasts (MEFs; 3–4 × 10^4^ cells/cm^2^) in 3i/LAF medium. The basal medium consisted of DMEM/F12 (Thermo Fisher Scientific, 10565‐018) and Neurobasal (Thermo Fisher Scientific, 21103‐049) (1:1) supplemented with 0.5% N2 (Thermo Fisher Scientific, 17502‐048), 1% B27 (Thermo Fisher Scientific, 12587‐010), 0.5% GlutaMAX (Thermo Fisher Scientific, 35050–061), 1% nonessential amino acids (Thermo Fisher Scientific, 11140‐050), 0.1 mm βmercaptoethanol (Thermo Fisher Scientific, 21985‐023), 1% penicillin–streptomycin (Thermo Fisher Scientific, 15140‐122), 5% Knockout serum replacement (KOSR, Thermo Fisher Scientific, A3181502), and 50µg/mL ascorbic acid (Sigma–Aldrich, A4544). The 3i/LAF medium was prepared by adding 1 µm CHIR99021 (Selleckchem, S1263), 2.5 µm IWR1endo (Selleckchem, S7086), 1 µm WH4023 (Selleckchem, S7565), 10 ng/mL recombinant human LIF (PeproTech, 300–05), 25 ng/mL Activin A (PeproTech, 120‐14E), and 10 ng/mL FGF2 (PeproTech, 100–18B). The ROCK inhibitor Y‐27632 (Selleckchem, S1049), 10 µm for passaging, 2 µm for maintenance, was used to support proliferation. Medium was replaced every day, and cells were passaged as single cells every 2–3 days using Accutase at a ratio from 1:3 to 1:5.

### 4.3 The Isolation of MEFs

Mouse fetuses (E13.5) were obtained from the uterus of pregnant mice and placed in a 10 cm dish containing DPBS (Gibco, C14190500BT) with 1% penicillin‐streptomycin. The torsos (without heads, limbs, tails, and viscera) of the fetuses were minced using sterilized surgical scissors and forceps, transferred into 1.5 mL centrifuge tubes, and digested with trypsin‐EDTA (Gibco, 25300120) in a water bath at 37°C for 5–10 min. The digestion was stopped by adding MEF medium (DMEM, Gibco, 11960‐044; supplemented with 10% FBS, Gibco, 16000–044, and 1% penicillin‐streptomycin). The cell suspension was transferred to a 10 cm plate and cultured at 37°C in an incubator with 5% CO_2_.

To prepare feeders for pgEpiSCs culture, the isolated MEFs were treated with mitomycin C (10 µg/mL, Selleckchem, S8146) for 2.5 h and washed three times with DPBS. The treated cells were then digested with trypsin‐EDTA for 5 min, neutralized with an equal volume of medium, and centrifuged at 1000 rpm for 5 min to harvest the cell pellets. Cells were frozen at a density of 2.4 × 10^6^ cells/mL. For use, they were thawed and seeded into culture wells before pgEpiSC inoculation.

### 4.4 Generation of SNL Feeders

The SNL cell line was kindly provided by Professor Zhonghua Liu's laboratory at Northeast Agricultural University (NEAU). The SNL feeders used for pPGCLCs culture need to be treated with mitomycin C for 2 h and washed three times with DPBS. Cells were digested for 5 min using trypsin‐EDTA, and an equal volume of medium was added for neutralization. They were centrifuged for 5 min at 1000 rpm to harvest the cellular precipitates. Cells can be frozen at a density of 2.4 × 10^6^ cells/mL, which can be thawed and inoculated into culture wells before inoculation with pPGCLCs.

### 4.5 Generation of the NANOS3‐tdTomato Knockin Reporter pgEpiSCs

For targeting of the NANOS3 locus to express tdTomato, male and female pgEpiSC lines were used. CRISPR–Cas9 technology was employed to design pGL3‐U6‐sgRNA‐PGK‐puromycin plasmid (Addgene, Cat# 51133, RRID: Addgene_51133) and construct a donor plasmid targeting the NANOS3 gene. The sgRNA target site was designed near the gene's stop codon. The oligonucleotide sequences for sgRNA were as follows:

Forward oligonucleotide (F): 5'‐caccgCCTAAGAGGCTGGCGCGAGC‐3';
Reverse oligonucleotide (R): 5'‐aaacGCTCGCGCCAGCCTCTTAGGc‐3'.

For the donor plasmid, 800 bp of genomic sequence flanking the stop codon was amplified to serve as the 5' and 3' homology arms for homologous recombination repair. After plasmid construction, both the sgRNA‐expressing plasmid and the donor plasmid were co‐transfected into pgEpiSCs using lipofection to achieve site‐specific labeling of the NANOS3 gene.

For transfection in a 6‐well plate format, feeder cells were seeded one day before. On the day of transfection, pgEpiSCs were dissociated with Accutase and passaged at a 1:3 ratio onto the feeder‐coated wells. After 8–12 h of culture, when cells were well attached, transfection was performed. Two 1.5 mL centrifuge tubes were prepared and labeled A and B. In tube A, 100 µL Opti‐MEM (Thermo Fisher Scientific, 31985070), 2 µg NANOS3‐donor plasmid, 2 µg pGL3‐U6‐sgRNA‐PGK‐puromycin plasmid, and 8 µL P3000 (Thermo Fisher Scientific, L3000075) reagent were gently mixed. In tube B, 100 µL Opti‐MEM and 3 µL Lipofectamine 3000 (Thermo Fisher Scientific, L3000075) were mixed, then combined with tube A and incubated at room temperature for 15 min to form transfection complexes. The original medium in the 6‐well plate was aspirated, and 2 mL of fresh pre‐warmed medium was added to each well. The transfection complexes (200 µL per well) were added dropwise, and the plate was gently swirled to mix.

At 12–14 h post‐transfection, the medium was replaced, and cells were allowed to recover for 24 h. Subsequently, the medium was replaced with 3i/LAF selection medium containing 0.3 µg/mL puromycin, which was changed daily for 3–4 days until complete cell death was observed in the control group (non‐transfected pgEpiSCs) and distinct colonies appeared in the experimental group. Colonies were marked under a microscope, picked using a pipette tip (mouth aspiration), and transferred to a 96‐well plate containing 20 µL of Accutase. After digestion at 37°C for 5 min, an equal volume of culture medium was added to terminate the reaction. Cells were dissociated into single cells or small clumps by pipetting and then transferred to a 48‐well plate for continued culture. When cells reached 80%–90% confluence, they were sequentially expanded to 24‐well and then 6‐well plates. During expansion, cells were collected for DNA extraction. The expected bands were amplified by PCR (forward primer, 5'‐AGAGTCCTATGGCCGCGAG‐3'; reverse primer, 5'‐GATCCGCGAAGGGTAGGTCG‐3') and subjected to Sanger sequencing to confirm the accuracy of the knock‐in. Finally, immunostaining confirmed that NANOS3‐tdTomato‐positive cells specifically expressed other PGC markers, indicating successful construction of the NANOS3 reporter cell line and its effectiveness in tracing PGCs differentiation.

### 4.6 Construction of Overexpression Vectors and Cell Lines

#### 4.6.1 Vector Construction and Gene Introduction

The pTRE3G‐[CDS]‐IRES‐Puro overexpression construct was generated as previously described [76]. In this study, the inducible overexpression vectors were constructed using homologous recombination cloning. For primer design, 15–30 bp homology arms complementary to the sequences flanking the linearized vector backbone were introduced at the 5' ends of the gene‐specific amplification primers. Target gene fragments were prepared by PCR amplification: using porcine testis cDNA as a template, the coding sequences of porcine SOX17, TFAP2C, and BLIMP1 were amplified with PrimeSTAR GXL DNA Polymerase (TaKaRa, R050A), a high‐fidelity DNA polymerase. The cDNA template was obtained by reverse transcription of RNA extracted from pooled porcine testis tissues. PCR products were examined by 1% agarose gel electrophoresis. After confirming correct band size, purity, and absence of primer dimers, gel blocks containing the target bands were excised under a UV transilluminator, transferred to clean 2.0 mL centrifuge tubes, and purified using the OMEGA Gel Extraction Kit (D2000‐02). The purified target gene fragments were assembled with the linearized vector backbone using NEBuilder HiFi DNA Assembly Master Mix (E2621L) via homologous recombination to complete the construction of TRE3G‐pSOX17, TRE3G‐pTFAP2C, and TRE3G‐pBLIMP1 vectors.

#### 4.6.2 Construction of Inducible Overexpression Cell Lines

NANOS3‐tdTomato pgEpiSCs were dissociated with Accutase and passaged at a 1:3 ratio. After 8–12 h of culture, when cells were well attached, transfection was performed. Two sterile 1.5 mL centrifuge tubes were prepared, each containing 100 µL of Opti‐MEM medium. In tube A, 2 µg of pCAG‐IRES‐Puro plasmid, 1 µg of TRE3G‐target gene vector, 1 µg of reverse tetracycline transactivator (rtTA) plasmid, and 8 µL of P3000 reagent were added and gently mixed. Tube B contained 100 µL of Opti‐MEM, and 3 µL of Lipofectamine 3000 reagent was added and gently mixed. The entire contents of tube A were transferred to tube B, mixed by gentle pipetting, and incubated at room temperature for 15 min to form transfection complexes. The original medium in the 6‐well plate was aspirated, and 2 mL of fresh pre‐warmed medium was added to each well. Transfection complexes (200 µL per well) were added dropwise, and the plate was gently swirled to mix. At 12–14 h post‐transfection, the medium was replaced, and cells were allowed to recover for 24 h. Subsequently, the medium was replaced with 3i/LAF selection medium containing G418 (100 µg/mL), which was changed daily for 6–8 days until complete cell death was observed in the untransfected control group and distinct colonies appeared in the experimental group. Single colonies were picked and expanded following the same method used for establishing the NANOS3 reporter cell line to obtain monoclonal cell lines. After induction with doxycycline (1 µg/mL for 24 h), the mRNA expression levels of the target genes were examined by qPCR to validate overexpression efficiency. Successfully validated cell lines were expanded and cryopreserved for subsequent differentiation experiments.

### 4.7 pPGCLCs Induction

All pPGCLCs induction experiments were performed in a humidified incubator with 5% CO_2_ at 37°C. pgEpiSCs were dissociated into single cells using Accutase and then seeded onto gelatin‐coated plates (Stem Cell Technologies, 07903). After incubation for 30 min at 37°C to allow feeder cells to attach to the gelatin‐coated surface at a density of 1.5 × 10^5^ cells/cm^2^, the non‐adherent pgEpiSCs were collected and counted. To induce pPGCLCs, 8000–10 000 cells per well of pgEpiSCs were plated with pPGCLCs induction medium (aRB27 medium which is composed of Advanced RPMI 1640 Medium (Thermo Fisher Scientific, 12633012) supplemented with 1% B27 supplement, 0.1 mm NEAA, 1% penicillin‐streptomycin, 1% GlutaMAX supplemented with 200 ng/mL BMP4 (Peprotech, 120‐05), 100 ng/mL SCF (PeproTech, 300‐071), 10 ng/mL LIF (Merck Millipore, LIF1010), 50 ng/mL EGF (PeproTech, AF‐100‐15), 2.5 µm IWR‐1‐endo (Selleck, S7086) and 10 µm Y‐27632 (Selleckchem, S1049) in a U‐bottom 96‐well plate (Greiner, 650185). For induction of exogenous transgenes, 1 µg/mL doxycycline was added to the medium. Note that 10% PVA (Sigma–Aldrich, P8136) was added for pPGCLCs induction from pgEpiSCs. At 8 days after pPGCLCs induction, pgEpiSCs aggregates were subjected to FACS (see ‘FACS analysis’) to isolate pPGCLCs.

As indicated in the description of each experiment, the following inhibitors and cytokines were added separately to the pPGCLCs differentiation medium: 10 ng/mL FGF2 (PeproTech, 100–18B), 25 ng/mL Activin A (PeproTech, 120‐14E), 10 µm bpV (HOpic) (Sigma–Aldrich, SML0884), 2.5 µm XAV939 (MedChemExpress, HY15147), 2.5 µm IWP2 (Selleckchem, S7085), 3 µm CHIR99021 (Selleckchem, S1263), 1 µm PD173074 (Selleckchem, S1264).

### 4.8 Fluorescence‐Activated Cell Sorting (FACS)

NANOS3‑tdTomato‑positive reporter cell lines were used as samples, while wild‑type pgEpiSCs subjected to the same differentiation protocol served as negative controls. NANOS3‐tdTomato‐positive aggregates obtained from differentiation were collected into 15 mL centrifuge tubes, centrifuged at 1000 rpm for 5 min, and the supernatant was discarded. Subsequently, 1 mL of trypsin‐EDTA was added, and digestion was performed in a 37°C water bath for 5 min. An equal volume of culture medium was added to terminate digestion, and cells were gently triturated to obtain a single‐cell suspension. After filtration through a 40 µm cell strainer, the suspension was centrifuged at 1000 rpm for 5 min. The supernatant was discarded, and the cell pellet was resuspended in FACS buffer, transferred to flow cytometry tubes, and kept on ice until use. Flow cytometry sorting was performed at the Flow Cytometry Facility of the Key Laboratory of Animal Genetics, Breeding and Reproduction, China Agricultural University. Collection tubes were pre‐loaded with 500 µL of aRB27 basal medium to receive the sorted NANOS3‐tdTomato positive cells, which were subsequently used for in vitro long‐term expansion culture or for constructing xenogeneic reconstituted ovaries or testes. Data obtained from sorting were analyzed using FlowJo software.

### 4.9 pPGCLCs Expansion Culture

SNL feeders were treated with mitomycin C (10 µg/mL) for 2.5 h, washed three times with DPBS, and then seeded into 12‐well plates at a density of 4.0 × 10^5^ cells per well in MEF medium. After culturing until 80%–90% confluence, the SNL feeder layers were ready for use. NANOS3‐tdTomato‐positive cells were isolated by fluorescence‐activated cell sorting (FACS) from either differentiation day 8 pPGCLCs or long‐term expanded pPGCLCs, and collected in ice‐cold FACS buffer. After centrifugation at 1000 rpm for 5 min, the supernatant was discarded, and cells were resuspended in long‐term expansion medium at a density of 5.0 × 10^4^ cells/mL. The original medium from SNL feeder layers was aspirated, and 1 mL of long‐term expansion medium (aRB27 basal medium supplemented with 10 µm Forskolin (Selleckchem, S2449), 20 ng/mL FGF2, 2.5 µm IWR1, 50 ng/mL EGF, 10 ng/mL LIF, and 50 ng/mL SCF) containing 10 µm Y‐27632 was added to each well. Plates were gently swirled and incubated at 37°C in a 5% CO_2_ incubator; this was designated as day 0. On day 1, an additional 1 mL of fresh long‐term expansion medium without Y‐27632 was added to each well. On day 3, the entire medium was replaced with 2 mL of fresh long‐term expansion medium without Y‐27632. Thereafter, medium changes were performed every other day, with monitoring of cell proliferation and fluorescence expression. When cells reached appropriate confluence for passaging (typically by day 10), the medium was aspirated, wells were washed with pre‐warmed DPBS, and Accutase was added for 5 min of digestion at 37°C. An equal volume of medium was added to terminate digestion, and cells were gently triturated to detach. The cell suspension was filtered through a 40 µm cell strainer, centrifuged at 1000 rpm for 5 min, and the supernatant was discarded. Cells were resuspended in fresh long‐term expansion medium containing Y‐27632 to initiate the next expansion cycle. Notably, FACS purification of NANOS3‐tdTomato‐positive cells was performed every 20 days (approximately every two passages) to maintain the germ cell‐like population. As indicated in the description of each experiment, the following inhibitors were added separately to the pPGCLCs expansion medium: Rolipram (Selleckchem, S1430).

### 4.10 Generation of Xenogeneic Reconstituted Ovaries and Testes

pPGCLCs were induced from pgEpiSCs. Xenogeneic reconstituted ovaries (xrOvaries) and xenogeneic reconstituted testes (xrTestes) were constructed by aggregating day 8 pPGCLCs with E13.5 fetal gonadal somatic cells from female or male embryos, respectively.

#### 4.10.1 Collection of pPGCLCs

Day 8 cell aggregates obtained from pgEpiSC induction were dissociated into single‐cell suspensions, and NANOS3‐tdTomato‐positive (NT^+^) cells (day 8 pPGCLCs) were isolated by flow cytometry as described in the Fluorescence‐activated cell sorting (FACS) section.

#### 4.10.2 Collection of Mouse Gonadal Somatic Cells

Referring to the method for human xrOvaries [77]. E13.5 mouse embryos were collected from pregnant Kunming white mice euthanized by cervical dislocation. Embryos were dissected in ice‐cold DPBS supplemented with 1% FBS and 1% penicillin/streptomycin. Fetal ovaries were identified based on morphological characteristics, and the mesonephros was removed using an insulin needle. Gonads from female and male embryos were collected separately. Intact gonads were transferred to 1.5 mL centrifuge tubes containing DPBS, centrifuged at 1000 rpm for 5 min, and the supernatant was discarded. Digestion was performed by adding 500 µL of Trypsin‐EDTA and incubating in a 37°C water bath for 5 min. An equal volume of MEF medium was added to terminate digestion, and the cell suspension was filtered through a 40 µm cell strainer, followed by centrifugation at 1000 rpm for 5 min to collect cells. The supernatant was discarded, and cells were resuspended in ice‐cold MACS buffer for counting. After another centrifugation, cells were resuspended in 80–100 µL of MACS buffer per 1 × 10^7^ total cells, and 20 µL of Anti‐SSEA1 microbeads (Miltenyi Biotec, 130‐094‐530) were added, gently mixed, and incubated on ice for 20–25 min with one gentle mix during incubation. Following incubation, cells were washed with 2–3 mL of MACS buffer, centrifuged at 1000 rpm for 5 min, and the supernatant was discarded. Cells were resuspended in 3–5 mL of MACS buffer. A separation column was placed in a magnetic field, rinsed with 500 µL of MACS buffer, and the cell suspension was slowly applied. The SSEA‐1‐negative cell fraction was collected as it flowed through. This step was repeated to obtain purified gonadal somatic cells. Collected cells were counted using an automated cell counter; a portion was used for xrOvaries and xrTestes construction, and the remaining cells were cryopreserved.

#### 4.10.3 Aggregation for xrOvaries or xrTestes

pPGCLCs and mouse gonadal somatic cells were mixed at a 1:10 ratio, with pPGCLCs at a density of 5 × 10^4^/mL and freshly isolated gonadal somatic cells at 5 × 10^5^/mL. If cryopreserved gonadal somatic cells were used, the density was adjusted to 7.5 × 10^5^/mL to compensate for cell loss during cryopreservation. The two cell suspensions were mixed in equal volumes and gently pipetted to ensure homogeneity. Aliquots of 200 µL of the mixed suspension (in aRB27 supplemented with 10 µm Y‐27632 and 50 ng/mL EGF) were added to U‐bottom 96‐well plates and incubated statically at 37°C in a 5% CO_2_ incubator for 48 h to allow cell aggregation. During this period, 400 µL of pre‐warmed xenogeneic reconstitution medium was added to the lower chamber of 24‐well Transwell‐COL inserts and equilibrated in the incubator for 1 h. After aggregation, well‐formed xenogeneic reconstituted ovary/testis aggregates were gently collected from the bottom of the U‐bottom plates using a mouth pipette and transferred onto Transwell‐COL membrane inserts (Corning, 3496) pre‐equilibrated with aRB27 medium containing 150 µm L‐ascorbic acid and 50 ng/mL EGF. Half‐medium changes were performed every 2 days. All xrOvaries and xrTestes were cultured at 37°C in a 5% CO_2_.

### 4.11 Growth Curve Analysis

pPGCLCs at a density of 5.0 × 10^4^ cells/mL were seeded into 24‐well plates pre‐coated with SNL feeder cells. At each indicated time point during the 10‐day culture period, the number of NT‐positive cells was recorded, with three replicate wells per time point. A cell growth curve was plotted using GraphPad Prism 8.0 software, with culture time (days) on the x‐axis and the corresponding number of viable cells on the y‐axis.

### 4.12 AP Staining

The AP staining assay kit (Millipore, SCR004) was performed exactly as recommended. The pgEpiSCs and their differentiated cells were fixed with 4% PFA for 5 min, and then washed with DPBS and incubated with AP staining working solution (A:B:DPBS = 2:1:1) for 15–20 min at 37°C in a 5% CO_2_ incubator protected from light. After washing with DPBS, cells were observed under a microscope.

### 4.13 Immunofluorescence Staining

#### 4.13.1 Immunofluorescence Staining in 4‐Well Plates

The culture medium was aspirated, and cells were gently washed twice with DPBS added gently along the well walls. Subsequently, 300 µL of 4% paraformaldehyde was added to each well, and cells were fixed at room temperature for 15 min, followed by three washes with DPBS on a shaker at low speed for 5 min each. Next, 300 µL of permeabilization solution (DPBS containing 0.1% Triton X‐100) was added to each well and incubated at room temperature for 15 min, followed by three washes with DPBS for 5 min each. Blocking solution (300 µL 3% BSA) was then added, and cells were blocked at room temperature for 2 h to reduce non‐specific binding. After aspirating the blocking solution, 300 µL of primary antibody working solution prepared in primary antibody diluent was added directly and incubated overnight at 4°C. The following day, the primary antibody was collected (stored at 4°C for future use), and cells were washed three times with DPBS for 5 min each. Subsequently, 300 µL of secondary antibody working solution prepared in secondary antibody diluent was added and incubated at room temperature for 30 min in the dark. Under dark conditions, cells were washed three times with DPBS for 5 min each. Finally, 300 µL of DAPI working solution was added and incubated at room temperature for 5 min in the dark. After three washes under dark conditions, 300 µL of DPBS was added to each well to prevent cell drying, and cells were ready for observation and imaging.

#### 4.13.2 Immunofluorescence Staining of Frozen Sections

Frozen sections were removed from the −80°C freezer and equilibrated at room temperature for 20–25 min. For fresh tissues, sections were immersed in 4% paraformaldehyde and fixed at room temperature for 15 min (this step can be omitted for previously fixed tissues). Sections were immersed in DPBS and washed three times on a shaker for 5 min each to remove residual OCT. A circle was drawn around the tissue using an immunohistochemistry pen, and 0.1% Triton X‐100 in PBS permeabilization solution was applied dropwise, followed by incubation at room temperature for 15 min. Sections were then washed three times with DPBS for 5 min each. Blocking solution (20–50 µL) was applied dropwise to completely cover the tissue, and sections were placed in a humidified chamber for blocking at room temperature for 1–2 h or overnight at 4°C. After aspirating the blocking solution, primary antibody working solution prepared in primary antibody diluent was applied dropwise, and sections were incubated in a humidified chamber overnight at 4°C (some antibodies may be incubated at 37°C for 2 h as an alternative). The following day, sections were immersed in DPBS and washed three times on a shaker for 5 min each. Secondary antibody working solution prepared in secondary antibody diluent was applied dropwise and incubated at room temperature for 30 min in the dark. Under dark conditions, sections were washed three times with DPBS for 5 min each. Finally, mounting medium containing DAPI was applied dropwise, and coverslips were placed gently to avoid air bubbles. The edges of the coverslips were sealed with nail polish. Sections were placed flat in a slide box and observed and imaged promptly using a microscope. All experiments included three biological replicates, and antibody information is provided in Table S7.

### 4.14 Frozen Section

Tissue samples were placed in 4% paraformaldehyde according to their size and fixed at 4°C for 4–24 h. After fixation, samples were washed three times with DPBS for 5 min each. Subsequently, tissues were sequentially dehydrated in 15% sucrose solution for 2–3 h, followed by overnight dehydration in 30% sucrose solution at 4°C (fresh tissues do not require fixation and dehydration treatment). Dehydrated tissues were placed into appropriately sized embedding molds, and pre‐cooled OCT (Sakura Finetek, 4583) compound (4°C) was added dropwise to completely cover the tissue, ensuring no air bubbles formed at the tissue‐OCT interface (if bubbles appeared, molds were placed at 4°C for 30 min to eliminate them). Using long forceps to hold the embedding mold, the bottom of the tissue was first immersed in liquid nitrogen for 10–15 s until the OCT turned white, then the entire block was fully immersed in liquid nitrogen until completely frozen, and subsequently transferred to a −80°C freezer for long‐term storage (for fresh tissues, dry ice should be used instead of liquid nitrogen to avoid tissue damage from excessively low temperatures). One hour before sectioning, the cryostat was turned on, with both chamber temperature and specimen head temperature set to ‐20°C. Tissue blocks were removed from −80°C and equilibrated in the cryostat for 10 min. OCT was applied to the specimen disk, and the tissue block was quickly mounted, then returned to the chamber for freezing. The blade, anti‐roll plate, and tissue angle were adjusted to ensure the tissue section plane was parallel to the blade edge. Rapid trimming was performed at 20 µm thickness until a complete tissue section plane was exposed, then section thickness was adjusted to 8–10 µm for slow, uniform sectioning to ensure continuous and complete sections. Sections were air‐dried for 1–5 min and either used directly for subsequent experiments or sealed and stored at −80°C.

### 4.15 qPCR and RNA‐seq Analysis (cDNA Amplification and qPCR)

Total RNA was extracted using the RNAprep pure Cell/Bacteria Kit RNAprep pure (TIANGEN, DP430), and its concentration was measured, which was then inverted to cDNA using Hifair III 1st Strand cDNA Synthesis SuperMix (YEASEN, 11141ES60) for qPCR. RT‐qPCR was performed using 2 × RealStar Green Power Mixture (GenStar, A311‐05). Primer information for RT‐qPCR was shown in Table S8. The resulting cycle thresholds (CT) were analyzed using the comparative CT (2^−ΔΔCT^) approach, and *GAPDH* was determined as an internal control. All experiments included three biological replicates, and primer sequences are shown in Table S8.

### 4.16 Bulk RNA‐seq Library Preparation, Data Processing and Analysis

Preparation of cell samples for sequencing. Cell samples with a minimum count of 5 × 10^6^ cells were prepared for sequencing. Cells were dissociated into single‐cell suspensions, centrifuged at 1000 rpm, and the supernatant was discarded. The cell pellet was washed once with DPBS, and the supernatant was aspirated again. Subsequently, 1 mL of Trizol solution was added to the cell pellet, mixed thoroughly by pipetting, and stored at −80°C. After all samples were collected, they were submitted to Novogene (Beijing, China) for RNA extraction, library construction, and sequencing. Upon completion of sequencing, Novogene delivered the raw data via a cloud server. After data integrity verification, subsequent transcriptomic analyses were performed, including gene expression quantification, screening of differentially expressed genes, and related functional enrichment analyses.

### 4.17 Sample Collection and Analysis for DNBelab C4 Single‐Cell RNA Sequencing

Cell samples with a minimum count of 1 × 10^5^ cells were prepared for single‐cell RNA sequencing. Cells were dissociated into single‐cell suspensions, centrifuged at 1000 rpm, and the supernatant was discarded. The cell pellet was resuspended in DPBS and filtered through a 40 µm cell strainer to remove cell clumps. Freshly prepared cell samples were submitted directly to Shanghai Majorbio Bio‐Pharm Technology Co., Ltd. for sequencing library construction. For cell samples of the same type collected at different time points, they were either stored temporarily at −80°C and transferred to liquid nitrogen for long‐term preservation the following day, or all samples were collected and submitted together to Majorbio for library construction. Upon completion of sequencing, Majorbio delivered the raw data via a cloud server. After quality control, subsequent single‐cell transcriptomic analyses were performed, including cell clustering, cell type annotation, differential gene expression screening, and related functional enrichment analyses.

### 4.18 Bulk RNA Sequencing Analysis

RNA‐seq raw data was processed by Trim Galore, clean reads were aligned to the reference genome of pig (Sus scrofa 11.1, Ensembl) with HISAT2 [78] and the expression was calculated by featureCounts [79] with Ensembl annotations (v112). Differentially expressed genes (DEGs) were identified using DESeq2 [80]. Additionally, to make a comparable analysis, batch effects and other unwanted variation were removed in the expression data using sva [81]. The GO and KEGG enrichment analysis of DEGs were performed using clusterProfiler [82].

### 4.19 Single‐Cell RNA Sequencing Analysis

Raw data analysis was performed using the DNBelab C Series HT scRNA analysis workflow (v3.0.0), which performs sequencing read alignment, aligned file manipulation, filtering, and feature counting with the genome of Sus scrofa 11.1 (ENSEMBL).

Gene expression were converted to a Seurat object using the Seurat v5 package (version 5.0.0) [83]. Cells were removed if they had >10 000 or fewer than 500 expressed genes or if over 10%–25% of their UMIs were derived from the mitochondrial genome. Mitochondrial genes were identified with the AnnotationHub package using Sus scrofa ENSEMBL v112. After filtering, the gene expression matrices for each sample were then log–normalized with scale factor 10 000 and the top 2000 variable features were identified. Immune system‐related anchors were identified, and the three datasets were integrated. The data were then scaled and linear dimension reduction was performed using principal component analysis (PCA) and uniform manifold approximation and projection (UMAP) for visualization.

To delineate the dynamic transitions of cell states during differentiation or development, we then performed single‐cell pseudo‐time trajectory analysis with the Seurat–analyzed object. One complementary computational approaches, Slingshot [84], was employed to robustly infer differentiation trajectories and pseudotemporal ordering of cells.

Marker gene selection and cluster annotation were performed as follows (Table S2). Differentially expressed genes for each cluster were identified using the FindAllMarkers function in Seurat with the following parameters: |log_2_FC| > 0.5, adjusted P < 0.05 (Bonferroni correction), and expression in >50% of cells within the cluster of interest. The resulting gene lists were manually curated against established lineage markers from the developmental biology literature.

Annotation reference datasets used in this study include published porcine fetal gonadal single‐cell transcriptomic datasets (E30–E35), which served as the primary reference. To complement the limited porcine reference data, human fetal gonadal single‐cell atlases (10–15 PCW) were used as additional cross‐species references. Detailed methods for gene set definition, module scoring, pseudobulk construction, and quantitative concordance assessment are provided below.

#### 4.19.1 Gene Set Definition and Scoring

To define in vivo developmental gene sets, we performed differential expression analysis on porcine fetal germ cell scRNA‐seq data. For the female lineage, we compared RA‐responsive oogonia with PGCs; for the male lineage, we compared pro‐spermatogonia with PGCs. Differentially expressed genes were identified using the Wilcoxon rank‐sum test with a threshold of adjusted *p* < 0.05. Genes were ranked by log_2_ fold change, and the top‐ranked genes were further filtered for known germ cell development‐related genes based on literature and database annotation. This yielded a female gene set of 296 genes and a male gene set of 77 genes. Gene set scores were calculated for each cell using the AddModuleScore function in Seurat, and the distribution of scores across cell states was visualized using UMAP embeddings and violin plots.

#### 4.19.2 Pseudobulk Construction

Single‐cell expression matrices were aggregated into pseudobulk samples according to sample origin and cell type annotation. The aggregated count matrix was normalized to counts per million (CPM), followed by log_2_(CPM + 1) transformation to stabilize variance.

#### 4.19.3 Quantitative Concordance Assessment of In Vivo and In Vitro Transitions

To assess how faithfully the in vitro induction system recapitulates in vivo developmental transitions, we calculated pseudobulk log_2_ fold changes for each gene in both the in vivo and in vitro transitions. Specifically, for the female lineage, the in vivo transition was RA‐responsive oogonia vs. PGCs, and the in vitro transition was oogonia‐like cells vs. PGCLCs. For the male lineage, the in vivo transition was pro‐spermatogonia vs. PGCs, and the in vitro transition was pro‐spermatogonia‐like cells vs. PGCLCs. Spearman correlation coefficients were calculated to quantify the overall concordance between in vivo and in vitro log_2_ fold changes, and linear regression was fitted. Key germ cell genes, including *STRA8*, *ZGLP1*, *DDX4*, and *SYCP3*, were annotated on the concordance scatter plots.

Cross‐platform integration and batch effect correction. To address platform‐specific differences between DNBelab C4 (this study) and 10x Genomics (reference datasets), we applied both Harmony and Reciprocal PCA (RPCA) integration algorithms. The performance of both methods was evaluated by assessing the mixing of datasets and the preservation of known biological structures. Downstream cross‐platform comparisons were based primarily on conserved marker gene expression patterns and relative transcriptomic similarities, and subtle quantitative differences between platforms were interpreted with caution.

Cross‐species ortholog mapping. One‐to‐one orthologs between Sus scrofa (Sscrofa11.1) and Homo sapiens (GRCh38) were retrieved from Ensembl BioMart. Only high‐confidence ortholog pairs were retained. For cross‐species integrative analyses, gene expression matrices were subset to shared orthologs prior to integration.

### 4.20 Downstream Analysis of scRNA Data

To investigate the biological functions of genes of interest, functional enrichment analyses were conducted based on the GO and KEGG databases. Then, to explore dynamic expression patterns of gene sets across conditions or pseudotime, genes were clustered based on their expression trends using the R package ClusterGVis [85]. Cell–cell communication analysis was performed using the R package CellChat [86]. The normalized gene expression matrix and cell type annotations derived from the Seurat object were used as input to infer intercellular signaling interactions. Since the ligand–receptor interaction database in CellChat is primarily curated for human and mouse, the human database provided in CellChat was used as a reference for pig. Orthologous genes between pig and human were mapped based on gene symbols to enable inference of potential signaling interactions. Communication probability between cell types was calculated using built‐in CellChat default parameters. All analyses were performed in R (version 4.3.1). Visualization was performed using ggplot2.

### 4.21 Statistical Analysis

The data used in this paper are shown as mean ± SEM and analyzed using Graphpad Prism 8.0. All analyses included at least three independent experiments. Statistical significance was calculated by *t* test or one‐way or two‐way ANOVA comparison on specific statement. n.s., non‐significant; ^*^ *p* < 0.05; ^**^ *p* < 0.01; ^***^ *p* < 0.001; ^****^ *p* < 0.0001.

## Author Contributions

J.H., T.C., and S.C. conceptualized this project and supervised the overall experiments. P.H. and T.C. generated the NANOS3‑tdTomato pgEpiSC reporter cell line. P.H. established the induction and long‑term expansion culture systems for oogonia‑like cells and pro‑spermatogonia‑like cells. L.Z., P.H., R.L., and D.G. performed bioinformatics analysis of transcriptomic and single‑cell transcriptomic data from different time points. P.H. and T.C. designed qPCR primers and carried out quantitative validation. P.H. performed sectioning of aggregates and immunofluorescence image processing. P.H., Y.W., and M.W. conducted flow cytometry analysis. M.Z. provided the raw data of female and male pgEpiSCs. P.H. and J.Z. established the SNL feeder cell line. P.H., Y.Y., Y.T., M.W., and F.Z. isolated fetal mouse gonad somatic cells. P.H. and X.C. prepared the experimental schematics. J.H., P.H., L.Z., and T.C., participated in manuscript writing, review, and editing.

## Funding

This work was supported by the National Key R&D Program of China (2022YFD1302201), the fellowship of China Postdoctoral Science Foundation (2026T190069, 2023M743802), the Biological Breeding‐National Science and Technology Major Project (2023ZD0407503), the Natural Science Foundation of China (32302746, 32370846), Pinduoduo‐China Agricultural University Research Fund (PC2024A01001), Hebei Medical Science Research Project (20210179). Hebei Traditional Chinese Medicine Research Project (2022186), the 2115 Talent Development Program of China Agricultural University, and High‐performance Computing Platform of China Agricultural University.

## Conflicts of Interest

The authors declare no conflicts of interest.

## Supporting information




**Supporting File 1**: advs78213‐sup‐0001‐SuppMat.docx.

**Supporting File 2**: advs78213‐sup‐0002‐TableS1.xlsx.

**Supporting File 3**: advs78213‐sup‐0003‐TableS2.xlsx.

**Supporting File 4**: advs78213‐sup‐0004‐TableS3.xlsx.

**Supporting File 5**: advs78213‐sup‐0005‐TableS4.xlsx.

**Supporting File 6**: advs78213‐sup‐0006‐TableS5.xlsx.

**Supporting File 7**: advs78213‐sup‐0007‐TableS6.xlsx.

**Supporting File 8**: advs78213‐sup‐0008‐TableS7.xlsx.

**Supporting File 9**: advs78213‐sup‐0009‐TableS8.xlsx.

## Data Availability

All data from this study have been deposited to GSA (https://ngdc.cncb.ac.cn/gsa) with the accession number CRA041744. For review purposes, temporary access is available via the following reviewer link: https://ngdc.cncb.ac.cn/gsa/s/Lidl2250. The data will be released upon publication of this manuscript. External single‐cell RNA sequencing datasets for human (GSE86146) and pig (CRA006365) are available through their respective accessions.
