# Supplementary material for: One‐Step Induction of Oogonia‐Like and Pro‐Spermatogonia‐Like Cells From Porcine Pluripotent Stem Cells
Source: Adv Sci (Weinh). 2026 Oct 11:e78213. Online ahead of print. doi: 10.1002/advs.78213 (PMC13655266; doi:10.1002/advs.78213)
Supplement: Supplementary file 1 [file ADVS-9999-e78213-s001.docx]

**One-step Induction and Long-term Expansion of Oogonia and Pro-spermatogonia from Porcine Pluripotent Stem Cells**

*Pengcheng He*^1, 5^, *Liang Zhong* ^2, 5^, *Tianzhi Chen*^1, 5, *^, *Yaxuan Yan*^1^, *Xinze Chen*^1^, *Runbo Li*^1^, *Yingjie Wang*^1^, *Yadi Teng*^1^, *Minglei Zhi*^1^, *Meng Wang*^1^, *Jinying Zhang*^1^, *Fan Zhao*^3^, *Dengfeng Gao*^4^, *Suying Cao*^3, *^, *Jianyong Han*^1, *^

^1^State Key Laboratory of Animal Biotech Breeding, Frontiers Science Center for Molecular Design Breeding (MOE), College of Biological Sciences, China Agricultural University, Beijing, China.

^2^Hebei Key Laboratory of Basic Medicine for Diabetes, Shijiazhuang Second Hospital, Hebei, China.

^3^Animal Science and Technology College, Beijing University of Agriculture, Beijing, China.

^4^State Key Laboratory of Swine and Poultry Breeding Industry, College of Animal Science and Technology, Sichuan Agricultural University, Chengdu, Sichuan, China.

^5^These authors contributed equally: Pengcheng He, Liang Zhong and Tianzhi Chen.

**Correspondence:** Tianzhi Chen ([chentianzhi15@163.com](http://chentianzhi15@163.com)), Suying Cao ([20137602@bua.edu.cn](http://20137602@bua.edu.cn)), Jianyong Han ([hanjy@cau.edu.cn](http://hanjy@cau.edu.cn)).

**Keywords:** primordial germ cells, pluripotent stem cells, porcine, pro-spermatogonia, oogonia, gametogenesis

**Figure S1. pgEpiSCs fail to differentiate directly into pPGCLCs.**

**(A)** Schematic diagram of the NANOS3-tdTomato reporter construction. **(B)** Genotyping of NANOS3-tdTomato cell lines. Homozygous lines show a single band, while heterozygous lines display double bands. **(C)** Male (Top) and female (Bottom) pgEpiSCs harboring the NANOS3-tdTomato reporter were both positive for AP staining. Scale bars: 50 μm. **(D)** IF staining for pluripotency markers NANOG, OCT4, SOX2, SSEA1, and TRA-1-81 in male pgEpiSCs. Scale bars: 20 μm. **(E)** IF staining for pluripotency markers NANOG, OCT4, SOX2, SSEA1, and TRA-1-81 in female pgEpiSCs. Scale bars: 20 μm. **(F)** Fluorescence microscope images of NANOS3-tdTomato reporter transgenic male pgEpiSCs cultured for 2, 4, 6, and 8 days in the presence of cytokines: BESLY (BMP4/EGF/SCF/LIF/Y-27632). Scale bars: 100 μm. **(G)** Fluorescence microscope images of NANOS3-tdTomato reporter transgenic female pgEpiSCs cultured for 2, 4, 6, and 8 days in the presence of cytokines: BESLY. Scale bars: 100 μm. **(H)** Flow cytometry analysis of NANOS3-tdTomato reporter transgenic male (Top) and female (Bottom) pgEpiSCs cultured for 8 days in the presence of cytokines: BESLY. **(I)** Box plot quantification of the percentage of NT^+^ cells in male and female aggregates at Day 8, as determined by flow cytometry. **(J)** RT-qPCR analysis of NANOS3-tdTomato reporter transgenic female pgEpiSCs cultured for 0, 2, 4, 6, and 8 days in the presence of cytokines BESLY. Values are normalized to *GAPDH* and presented as mean ± SD relative to untreated day 0 controls, ns, *P* > 0.05, **P* < 0.05, ***P* < 0.01, ****P* < 0.001.

**Figure S2. Molecular characterization of pgEpiSCs during transcription factor-activated differentiation.**

**(A)** Fluorescence microscope images of NANOS3-tdTomato reporter transgenic male pgEpiSCs cultured for 2, 4, 6, and 8 days with induction of TFAP2C in the presence of cytokines: BESLY. Scale bars: 100 μm. Biologically independent experiments, n = 3 for TFAP2C are shown. **(B)** Fluorescence microscope images of NANOS3-tdTomato reporter transgenic male pgEpiSCs cultured for 2, 4, 6 and 8 days with induction of BLIMP1 in the presence of cytokines: BESLY. Scale bars: 100 μm. Biologically independent experiments, n = 3 for BLIMP1 are shown. **(C)** Flow cytometry of NANOS3-tdTomato reporter transgenic male pgEpiSCs cultured for 8 days with induction of TFAP2C,and BLIMP1 in the presence of cytokines: BESLY. **(D)** RT-qPCR analysis of gene expression male pgEpiSCs induced for 8 days with TFAP2C and BLIMP1 transgenes in the presence of cytokines: BESLY. Values are normalized to *GAPDH* and presented as mean ± SD relative to untreated day 0 controls. **(E)** Heatmap of lineage-specific gene expression in male pgEpiSCs and female pgEpiSCs. Genes are grouped by functional lineage (core pluripotency, PGC). Color scale represents relative expression levels. **(F)** IF analyses of the expression of TFAP2C, BLIMP1, OCT4, NANOG, SOX17, and SOX2 in NT^+^ (red) cells of aggregates induced from transgenic male pgEpiSCs after 8 days induction of SOX17. The cells were counterstained with DAPI (gray), and merged images are shown on the right. Scale bars: 20 μm. **(G)** IF analyses of the expression of TFAP2C, BLIMP1, OCT4, NANOG, SOX17, and SOX2 in NT^+^ (red) cells of aggregates induced from transgenic female pgEpiSCs after 8 days induction of SOX17/TFAP2C. The cells were counterstained with DAPI (gray), and merged images are shown on the right. Scale bars: 20 μm. **(H)** RT-qPCR analysis of gene expression in NANOS3-tdTomato-positive cells derived from female pgEpiSCs induced for 8 days with SOX17, SOX17/TFAP2C, or SOX17/BLIMP1 transgenes in the presence of cytokines: BESLY. Values are normalized to *GAPDH* and presented as mean ± SD relative to untreated day 0 controls. Biologically independent experiments, n = 3 for day 0 and day 8.

**Figure S3. Transcriptomic comparison of pPGCLCs and *in vivo* PGCs.**

**(A)** Fluorescence microscope images and flow cytometry of NANOS3-tdTomato reporter transgenic male pgEpiSCs cultured for 8 days with induction of SOX17 in the presence of cytokines: control (no cytokines), BESLY (BMP4/EGF/SCF/LIF/Y-27632), BESLY+FGF2, BESLY + PD173074, BESLY + Activin A, BESLY + bpV. Scale bars: 100 μm. **(B)** Bar graph quantification of the percentage of NANOS3-tdTomato-positive cells in pgEpiSCs (Control, no cytokines) and Day 8 aggregates under the indicated treatments. Statistical significance was determined by t-test: ns, *P* > 0.05, **P* < 0.05, ***P* < 0.01, ****P* < 0.001. Error bars represent mean ± SEM. Biologically independent experiments, n = 3. **(C)** Hierarchical clustering of transcriptomic data from pgEpiSCs and aggregates at days 2, 4, 6, and 8 of differentiation, with or without IWR1 treatment. Euclidean distance was used for clustering. **(D)** Bar graph showing the number of differentially expressed genes (DEGs) at days 2, 4, 6, and 8 of pPGCLC differentiation, comparing IWR1-treated and untreated samples. **(E)** Gene Ontology (GO) enrichment analysis of upregulated (red) and downregulated (blue) genes in IWR1-treated aggregates at day 8, relative to untreated controls. **(F)** Sample correlation heatmap of male and female pgEpiSCs and PGCLCs, based on global gene expression. Euclidean distance was used for hierarchical clustering. Color scale indicates Pearson correlation coefficient. **(G)** Principal component analysis (PCA) of transcriptomic data from male and female pgEpiSCs and PGCLCs. **(H)** Heatmap of expression levels for key germ cell and pluripotency-related genes in porcine Ectoderm (E11), PGCs (E14, E31), female/male pgEpiSCs and female/male PGCLCs (8 days). Color scale indicates relative expression. **(I)** Heatmap of selected PGC and pluripotency marker genes across male and female pgEpiSCs and PGCLCs.

**Figure S4. Supplementary single-cell transcriptomic profiling of pro-spermatogonia and oogonia-like during differentiation.**

**(A, B)** Quality control metrics of RNA-seq data from male **(A)** and female **(B)** samples, including feature counts, RNA counts, and mitochondrial RNA percentage, at key stages: pgEpiSCs, day 2, day 4, and day 8 aggregates. **(C, D)** UMAP visualization of cell population transitions during differentiation in male **(C)** and female **(D)** pgEpiSCs and aggregates at days 2, 4, and 8. Cells are colored by annotated cell types. **(E)** UMAP visualization of representative genes for male PGC and gonadal somatic cells. Expression levels were color–coded. **(F)** UMAP visualization of representative genes for female PGC and gonadal somatic cells. Expression levels were color–coded.

**Figure S5.** **Molecular Characteristics of Germ Cells and Gonadal Somatic Cells.**

**(A, B)** Gene expression heatmaps with Gene Ontology (GO) enrichment analysis for female **(A)** and male **(B)** germ cell lineages. Heatmaps show z-score normalized expression across cell populations (pgEpiSCs, Pre-PGCLCs, PGCLCs, pro-spermatogonia/oogonia-like). **(C)** IF analyses of the expression of SOX9 (yellow) in NT^+^ (red) cells of aggregates induced from transgenic male pgEpiSCs after 8 days induction of SOX17 in the presence of cytokines: BMP4/EGF/SCF/LIF/IWR1/Y-27632, Scale bars: 50 μm (zoomed-out), 20 μm (zoomed-in). **(D)** IF analyses of the expression of KRT19 (yellow) in NT^+^ (red) cells of aggregates induced from transgenic male pgEpiSCs after 8 days induction of SOX17 in the presence of cytokines: BMP4/EGF/SCF/LIF/IWR1/Y-27632, Scale bars: 50 μm (zoomed-out), 20 μm (zoomed-in). **(E, F)** Gene expression heatmaps with GO enrichment analysis for male **(E)** somatic cell lineages (Sertoli-like, GPCs-like, epithelial cells-like) and female **(F)** somatic cell lineages (granulosa cell-like, stromal cells-like, GPCs-like). **(G, H)** Violin plots showing the expression levels of genes involved in PRC2 complex, MII Complex and Chromatin Remodeller across four cell types: pgEpiSCs, Pre-PGCLCs, PGCLCs, and Oogonia-like **(G)** or Pro-spermatogonia-like **(H)**.

**Figure S6.** **Cross-species and *in vivo* concordance analysis of female and male *in vitro*-derived germ cells.**

**(A)** Integrative analysis of female pgEpiSC-derived PGCLCs and oogonia-like cells with porcine fetal RA-responsive oogonia, showing UMAP visualization of integrated cell populations (colored by annotated cell types) and representative gene expression (color-coded by expression level). **(B)** Expression trends of selected germ cell genes along the pseudotime trajectory from female pgEpiSC-derived pPGCLCs to oogonia-like cells. Early PGC markers are expressed in oogonia-like cells, while RA-responsive oogonia markers *STRA8* and *ZGLP1* remain uninduced. **(C)** Integrative analysis of male pgEpiSC-derived PGCLCs and pro-spermatogonia-like cells with porcine fetal pro-spermatogonia, showing UMAP visualization of integrated cell populations (colored by annotated cell types) and representative gene expression (color-coded by expression level). **(D)** Expression trends of selected germ cell genes along the pseudotime trajectory from male pgEpiSC-derived pPGCLCs to pro-spermatogonia-like cells. Both early PGC markers and late germ cell markers show similar trends between *in vitro* and *in vivo* populations. **(E)** UMAP of female gene-set score (296 marker genes), color-coded by module score. **(F)** Violin plots of gene-set scores across PGCs, PGCLCs, RA-responsive oogonia, and oogonia-like cells. **(G)** Concordance of mean gene expression (log₂CPM) between *in vivo* and *in vitro* female germ cells. **(H)** Pseudobulk log₂ fold-change concordance between the *in vivo* transition (RA-responsive oogonia vs. PGCs) and the *in vitro* transition (oogonia-like cells vs. PGCLCs); key genes (e.g., *STRA8*, *ZGLP1*, *DDX4*, *SOX17*, *TFAP2C*) are highlighted. In (G) and (H), dashed line = y = x; red/grey line = linear regression; Spearman r and P values are shown. **(I)** Bubble plot of gene-set log₂ fold changes across *in vivo* groups (E30-PGC, E30-RA oogonia, E35-PGC, E35-RA oogonia) and *in vitro* groups (PGCLCs and oogonia-like cells at various time points); bubble size indicates gene-set log₂FC and color indicates comparison type. **(J)** UMAP of male gene-set score (77 marker genes), color-coded by module score. **(K)** Violin plots of gene-set scores across PGCs, PGCLCs, pro-spermatogonia-like cells, and pro-spermatogonia. **(L)** Concordance of mean gene expression (log₂CPM) between *in vivo* and *in vitro* male germ cells. **(M)** Pseudobulk log₂ fold-change concordance between the *in vivo* transition (pro-spermatogonia vs. PGCs) and the *in vitro* transition (pro-spermatogonia-like cells vs. PGCLCs); key genes (e.g., *SYCP3*, *DDX4*, *PIWIL4*, *SOX17*, *STRA8*, *NANOG*, *BLIMP1*, *NANOS3*, *TFAP2C*) are highlighted. In (L) and (M), dashed line = y = x; red/grey line = linear regression; Spearman r and *P* values are shown. **(N)** Bubble plot of gene-set log₂ fold changes across *in vivo* groups (E24-PGC, E27-PGC, E30-PGC, E35-PGC, E35-pro-spermatogonia) and *in vitro* groups (PGCLCs and pro-spermatogonia-like cells at various time points); bubble size indicates gene-set log₂FC and color indicates comparison type.

**Figure S7. Transcriptomic characterization of gonadal somatic-like cells and germ cell marker expression across developmental stages.**

**(A)** Integrative analysis of male pgEpiSC-derived PGCLCs and pro-spermatogonia-like cells with porcine fetal pro-spermatogonia, showing UMAP visualization of integrated cell populations (colored by annotated cell types) and representative gene expression (color-coded by expression level). **(B)** Integrative analysis of female pgEpiSC-derived gonadal somatic-like cells with porcine fetal ovarian somatic cells, showing UMAP visualization of integrated cell populations (colored by annotated cell types) and representative gene expression for granulosa and stromal cells (color-coded by expression level). **(C)** Dot plots displaying the expression of key marker genes across female pgEpiSC-derived Granulosa cell-like, Stromal cell-like and GPC-like, together with porcine female fetal Granulosa cell, Stromal cell and GPC, with dot size representing the percentage of cells expressing the gene and color intensity representing expression level. **(D)** Dot plots displaying the expression of key marker genes across male pgEpiSC-derived Sertoli cell-like and GPC-like, together with porcine male fetal Sertoli cell and GPC, with dot size representing the percentage of cells expressing the gene and color intensity representing expression level. **(E)** Dot plots displaying the expression of key marker genes across male pgEpiSC-derived PGCLCs, Oogonia-like cells, and human female fetal germ cells (PGC, RA responsive Oogonia and Meiotic Oogonia), with dot size representing the percentage of cells expressing the gene and color intensity representing expression level.

**Figure S8. Ligand-receptor analysis of germ cells and gonadal somatic cells.**

**(A)** Dot plots showing ligand–receptor interactions in Day 2 (left) and Day 4 male (right) aggregates. Each dot represents a ligand–receptor pair, with color intensity indicating expression level. **(B)** Dot plots showing ligand–receptor interactions in Day 2 (Top) and Day 8 female (Bottom) aggregates. Each dot represents a ligand–receptor pair, with color intensity indicating expression level. **(C, D)** Heatmaps summarizing the importance of different signaling components in female **(C)** and male **(D)** aggregates. Color intensity reflects the relative importance score of each cell type in mediating pathway activity.

**Figure S9. Analysis of pPGCLCs and gonadal somatic cells under different concentrations of EGF treatment.**

**(A)** IF analyses of the expression of SOX9 (green) and GATA4 (purple) in NT^+^ (red) cells of aggregates induced from transgenic male pgEpiSCs after 8 days induction of SOX17 in the presence of cytokines: BMP4/SCF/LIF/IWR1/Y-27632+EGF doses (20 ng, 50 ng, 100 ng, and 200 ng). The cells were counterstained with DAPI (blue), Scale bars: 50 μm (zoomed-out), 20 μm (zoomed-in). **(B)** IF analyses of the expression of KRT19 (green) and GATA4 (purple) in NT^+^ (red) cells of aggregates induced from transgenic in male pgEpiSCs with 8 days induction of SOX17 in the presence of cytokines: BMP4/SCF/LIF/IWR1/Y-27632+EGF doses (20 ng, 50 ng, 100 ng, and 200 ng). The cells were counterstained with DAPI (blue), Scale bars: 50 μm (zoomed-out), 20 μm (zoomed-in). **(C)** Quantification of immunostaining data in Figure S8A. The numbers of Other, SOX9^-^GATA4^+^, SOX9^+^GATA4^+^, SOX9^+^GATA4^-^ and NT^+^ induced from transgenic pgEpiSCs after 8 days induction of SOX17 in the presence of cytokines: BMP4/SCF/LIF/IWR1/Y-27632+EGF doses (20 ng, 50 ng, 100 ng, and 200 ng). **(D)** Quantification of immunostaining data in Figure S8B. The numbers of Other, KRT19^+^GATA4^+^, KRT19^+^GATA4^-^, and NT^+^ induced from transgenic pgEpiSCs with 8 days induction of SOX17 in the presence of cytokines: BMP4/SCF/LIF/IWR1/Y-27632+EGF doses (20 ng, 50 ng, 100 ng, and 200 ng). **(E)** IF analyses of the expression of KRT19 (green) and GATA4 (purple) in NT^+^ (red) cells of aggregates induced from transgenic male pgEpiSCs after 8 days of induction SOX17 in the presence of cytokines: EGF/SCF/LIF/IWR1/Y-27632+BMP4 doses (50 ng, 100 ng, 200 ng, and 500 ng). The cells were counterstained with DAPI (blue), Scale bars: 50 μm (zoomed-out), 20 μm (zoomed-in). **(F)** Quantification of immunostaining data in Figure 6I. The numbers of Other, SOX9^-^GATA4^+^, SOX9^+^GATA4^+^, SOX9^+^GATA4^-^ and NT^+^ induced from transgenic pgEpiSCs with 8 days induction of SOX17 in the presence of cytokines: EGF/SCF/LIF/IWR1/Y-27632+BMP4 doses (50 ng, 100 ng, 200 ng, and 500 ng). **(G)** Quantification of immunostaining data in Figure S8E. The numbers of Other, , KRT19^+^GATA4^+^, KRT19^+^GATA4^-^ and NT^+^ induced from transgenic pgEpiSCs with 8 days induction of SOX17 in the presence of cytokines: EGF/SCF/LIF/IWR1/Y-27632+BMP4 doses (50 ng, 100 ng, 200 ng, and 500 ng).

**Figure S10. Analysis of xenogeneic reconstituted ovaries and testes following *in vitro* culture.**

**(A)** Fluorescence microscopy images of xrOvaries. NT^+^ cells, derived from male pgEpiSCs following induction with SOX17 for 8 days, were aggregated with mouse E13.5 gonadal somatic cells. The aggregates were cultured in aRB27 basal medium supplemented with EGF for 21 days. Scale bars: 200 μm. **(B)** IF staining of xrOvaries at day 21 of culture showing NT^+^ (red, male) and DDX4 (green) expression. The cells were counterstained with DAPI (blue), and merged images are show on the right. Scale bars: 50 μm (zoomed-out), 20 μm (zoomed-in). **(C)** IF staining of xrOvaries at day 21 of culture showing NT^+^ (red, female) and DDX4 (green) expression. The cells were counterstained with DAPI (blue), and merged images are shown on the right. Scale bars: 50 μm (zoomed-out), 20 μm (zoomed-in). **(D)** IF staining of xrOvaries at day 21 of culture showing NT^+^ (red, female), DAZL (green) and FOXL2 (purple) expression. The cells were counterstained with DAPI (blue), and merged images are shown on the right. Scale bars: 50 μm (zoomed-out), 20 μm (zoomed-in). **(E)** HE staining of xrTestes at days 7, 14, and 21 of culture. Scale bars: 100 μm (zoomed-out), 50 μm (zoomed-in).

**Figure S11. Mouse and human medium fail to support the long-term expansion of pPGCLCs.**

**(A)** Fluorescence microscopy images of NANOS3-tdTomato-positive pPGCLCs sorted on day 8 and expanded for 2, 4, and 10 days in mouse media (GK15 + 2.5% FBS + Forskolin/Rolipram/SCF). Scale bars: 100 μm. **(B)** Fluorescence microscopy images of NANOS3-tdTomato-positive pPGCLCs sorted on day 8 and expanded for 2, 4, and 10 days in human media (GK15 + 2.5% FBS + Forskolin/FGF2/SCF). Scale bars: 100 μm. **(C)** Flow cytometry analysis of NANOS3-tdTomato-positive cells in Long-term expanded pPGCLCs in mouse medium or human medium for 10 days. **(D)** IF analyses of the expression of SOX2, SOX17, and TFAP2C (green) in NT^+^ (red) cells during NT^+^ cell expansion culture in mouse medium. These cells were counterstained with DAPI (blue). Scale bars: 50 μm. **(E)** IF analyses of the expression of SOX2, SOX17 and TFAP2C (green) in NT^+^ (red) cells during NT^+^ cell expansion culture in human medium. These cells were counterstained with DAPI (blue). Scale bars: 50 μm. **(F)** RT-qPCR analysis of gene expression in pgEpiSCs, pPGCLCs at differentiation day 8, and pPGCLCs after 2 days and 10 days of long-term culture. Values are normalized to *GAPDH* and presented as mean ± SD relative to untreated pgEpiSC controls.

**Figure S12. Comparison of the effects of different BMP cytokines (BMP2, BMP4, BMP7) on the long-term expansion of pPGCLCs.**

**(A)** Heatmap showing unsupervised hierarchical clustering of differentially expressed genes across human and mouse medium (including aRB27, GK15, NBK5, BMP4/2/7 conditions). Rows represent genes, columns represent samples. Color intensity indicates relative expression level. **(B)** Bar chart summarizing the number of differentially expressed genes (DEGs) under different comparisons: Human-aRB27 vs Human-GK15, Human-aRB27 vs Human-NBK5, and Human-aRB27 vs Mouse-aRB27. Red bars indicate upregulated genes, blue bars indicate downregulated genes. **(C)** Bar charts displaying Gene Ontology (GO) enrichment results for DEGs from two comparisons: (top) Human-aRB27 vs Mouse-aRB27, (bottom) Human-aRB27 vs Human-GK15. Blue bars represent downregulated GO terms, red bars represent upregulated GO terms. **(D)** Bubble plot showing Gene Set Enrichment Analysis (GSEA) results for the Human-aRB27 vs Human-GK15 comparison.

**Figure S13. Comparison of the effects of different BMP cytokines (BMP2, BMP4, BMP7) on the long-term expansion of pPGCLCs.**

**(A)** PCA plot of pPGCLCs expanded for 10 days under various culture conditions. Transcriptomic clustering of pPGCLCs cultured for 10 days in expansion medium supplemented with different BMP cytokines (BMP2, BMP4, and BMP7). **(B)** Box plots showing changes in gene expression levels of pPGCLCs cultured for 10 days in expansion medium supplemented with different BMP cytokines (BMP2, BMP4, and BMP7). **(C)** Volcano plots visualizing gene expression changes in three BMP-related comparisons: Add BMP4 vs No BMP, Add BMP7 vs No BMP, and Add BMP2 vs No BMP. Red points indicate upregulated genes, blue points indicate downregulated genes. **(D)** Bubble plot showing GSEA results for the Add BMP4 vs No BMP comparison. **(E)** IF staining of pPGCLCs cultured for 10 days in expansion medium supplemented with BMP2, BMP4, or BMP7. NT^+^ (red) and DDX4 (cyan) expression are shown, with DAPI (blue) counterstaining for nuclei. Scale bars: 20 μm. **(F)** Growth curves of pPGCLCs cultured for 30 days in medium without BMP supplementation (Control) or supplemented with BMP2, BMP4, or BMP7. The number of pPGCLCs was quantified based on NANOS3-tdTomato-positive cells. **(G)** Growth curves of pPGCLC-derived cells induced from the indicated pgEpiSC lines. The number of pPGCLC-derived cells was quantified based on NANOS3-tdTomato-positive cells. **(H)** IF analyses of the expression of DAZL (green) in NT^+^ (red) cells during NT^+^ cell expansion culture 30 days at Female (Top) and Male (bottom). Scale bars: 20 μm.

**Figure S14. Molecular characterization of pro-spermatogonia-like cells under long-term expansion culture.**

**(A)** RT-qPCR analysis of gene expression in pgEpiSCs, day 8 pPGCLCs, and pPGCLCs after 10, 30, 60, and 120 days of long-term expansion culture. Values are normalized to *GAPDH* and presented as mean ± SD relative to untreated day 0 controls. **(B)** Bar plots showing the proportions of PGCLCs and pro-spermatogonia-like cells at each time point (day 80, 100, and 120) of long-term expansion. **(C)** Pseudotemporal analysis of the trajectory from pgEpiSCs to pro-spermatogonia-like cells following 120 days of long-term expansion. **(D)** Pseudotime trajectory analysis of gene expression changes for pluripotency (*OCT4, NANOG, SOX2,* and *OTX2*) markers. **(E)** Violin plots showing the expression levels of key germline and pluripotency genes across multiple cell types (pgEpiSCs, PGCLCs, pro-spermatogonia-like cells (ProSLCs) at 8/30/100/120 days). **(F)** Violin plots showing the expression levels of DNA methylation/demethylation and histone modification-related genes across multiple cell types (pgEpiSCs, PGCLCs, pro-spermatogonia-like cells (ProSLCs) at 8/30/100/120 days).
